# Supplementary material for: Therapeutic targeting of circ‐CUX1/EWSR1/MAZ axis inhibits glycolysis and neuroblastoma progression
Source: EMBO Mol Med. 2019 Nov 11;11(12):e10835. doi: 10.15252/emmm.201910835 (PMC6895612; doi:10.15252/emmm.201910835)
Supplement: Supplementary file 1 — Appendix [file EMMM-11-e10835-s001.pdf]

Therapeutic targeting of *circ-CUX1*/EWSR1/MAZ axis inhibits glycolysis and neuroblastoma progression

Huanhuan Li, Feng Yang, Anpei Hu, Xiaojing Wang, Erhu Fang, Yajun Chen, Dan Li, Huajie Song, Jianqun Wang, Yanhua Guo, Yang Liu, Hongjun Li, Kai Huang, Liduan Zheng, Qiangsong Tong

Running title: *circ-CUX1*/EWSR1/MAZ axis in glycolysis

Key words: circular RNA/CUT-like homeobox 1/EWS RNA binding protein 1/MYC-associated zinc finger protein/tumor progression

Table of contents

Appendix Figure S1. CUX1 regulates aerobic glycolysis in NB cells.

Appendix Figure S2. Roles and expression profiles of *CUX1* and target genes in tumors.

Appendix Figure S3. Expression of *CUX1*, *ENO1*, *GPI*, or *PGK1* is associated with survival of tumor patients.

Appendix Figure S4. Copy number and genetic variants of *CUX1* gene in human cancers.

Appendix Figure S5. Specific ectopic expression or knockdown of *circ-CUX1* and *CUX1* in tumor cells.

Appendix Figure S6. *circ-CUX1* regulates aerobic glycolysis and EWSR1-MAZ interaction.

Appendix Figure S7. Expression profiles of MAZ and downstream target genes.

Appendix Figure S8. *Circ-CUX1* facilitates EWSR1-mediated MAZ transactivation in NB cells.

Appendix Figure S9. Gene knockdown and effects of EIP-22 on NB cells *in vitro.*

Appendix Figure S10. EIP-22 inhibits tumorigenesis and aerobic glycolysis of NB cells *in vivo.*

Appendix Figure S11. Lentivirus-mediated *circ-CUX1* knockdown inhibits aerobic glycolysis and tumor progression.

Appendix Table S1. Primer sets used for RT-PCR, qRT-PCR, RIP, PCR and ChIP.

Appendix Table S2. Oligonucleotide sets used for constructs, guide DNA, and probes.

Appendix Table S3. Oligonucleotides encoding short hairpin RNAs.

Appendix Table S4. *P* values for differences between experimental groups in figures.

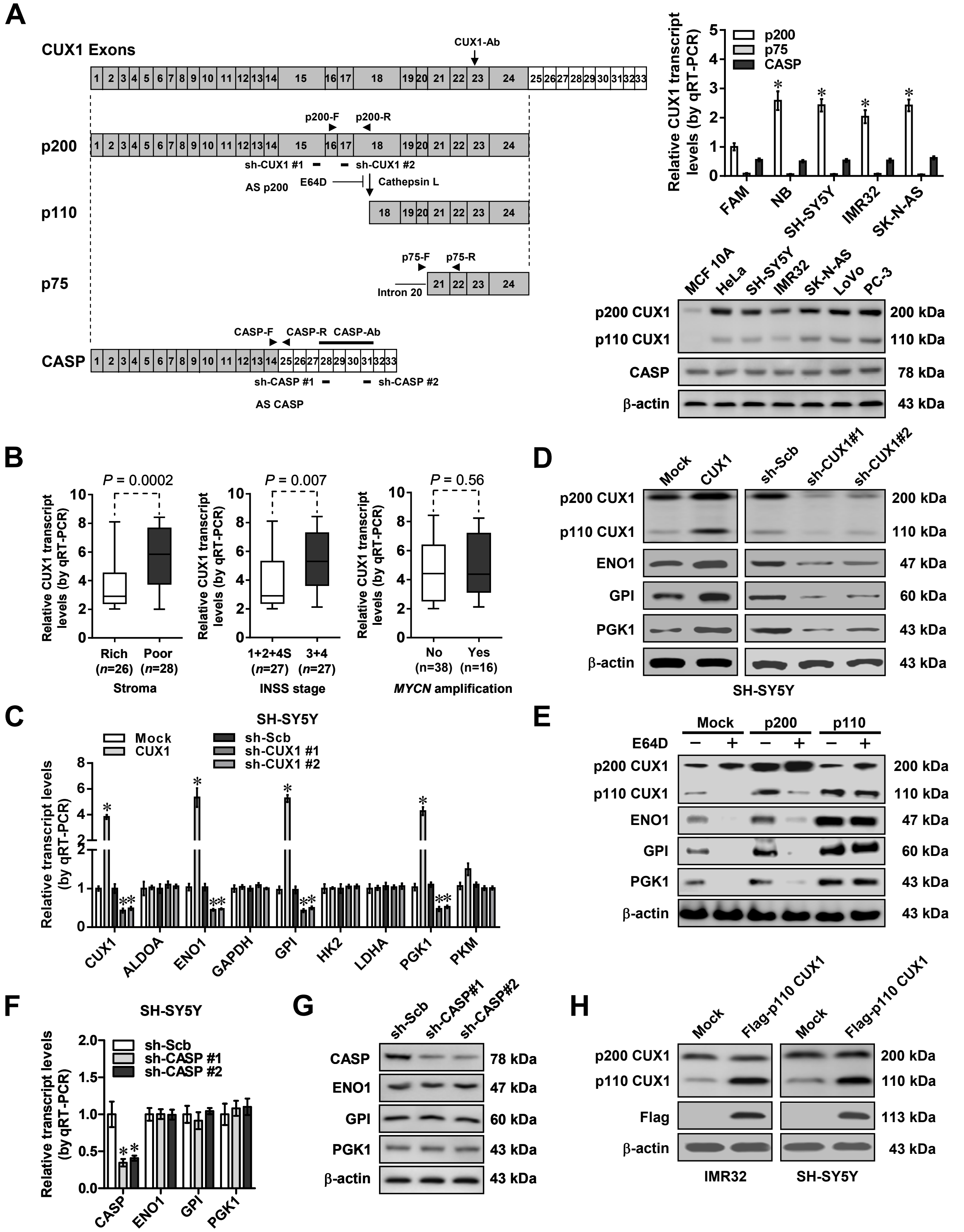

Appendix Figure S1. CUX1 regulates aerobic glycolysis in NB cells. A, Real-time qRT-PCR (right upper panel) and western blot (right lower panel) assays showing the expression of *CUX1* isoforms in fetal adrenal medulla (FAM, *n*=13), NB tissues (*n*=54), non-transformed normal MCF 10A cells, and tumor cell lines, using antibodies and primers as indicated (left panel). One-way ANOVA, * *P*<0.05 vs. FAM. **B**, Real-time qRT-PCR assay indicating *p200 CUX1* levels (normalized to β-actin) in NB tissues (*n*=54) with different stroma content, INSS stages, or *MYCN*-amplification status. Bars are means and whiskers (min to max). Student's *t*-test, *P*=0.0002, *P*=0.007, and *P*=0.56. **C** and **D**, Real-time qRT-PCR (C, normalized to β-actin, *n*=5) and western blot (D) assays revealing the expression of *CUX1* and glycolytic genes in SH-SY5Y cells stably transfected with empty vector (mock), *p200 CUX1*, scramble shRNA (sh-Scb), sh-CUX1 #1, or sh-CUX1 #2. Student's *t*-test, one-way ANOVA, * *P*<0.05 vs. mock or sh-Scb. **E**, Western blot assay indicating the expression of *CUX1* and glycolytic genes in IMR32 cells transfected with mock, *p200*, or *p110*, with or without E64D (10 μmol·L^{-1}) treatment for 24 hrs. **F** and **G**, Real-time qRT-PCR (F, normalized to β-actin, *n*=5) and western blot (G) assays showing the expression of *CASP* and glycolytic genes in SH-SY5Y cells transfected with sh-Scb, sh-CASP #1, or sh-CASP #2. One-way ANOVA, * *P*<0.05 vs. sh-Scb. **H**, Western blot assay indicating the expression of *CUX1* in IMR32 and SH-SY5Y cells stably transfected with mock or Flag-tagged *p110 CUX1*. Data information: Data are presented as mean ± SEM. Exact *P* values are specified in Appendix Table S4.

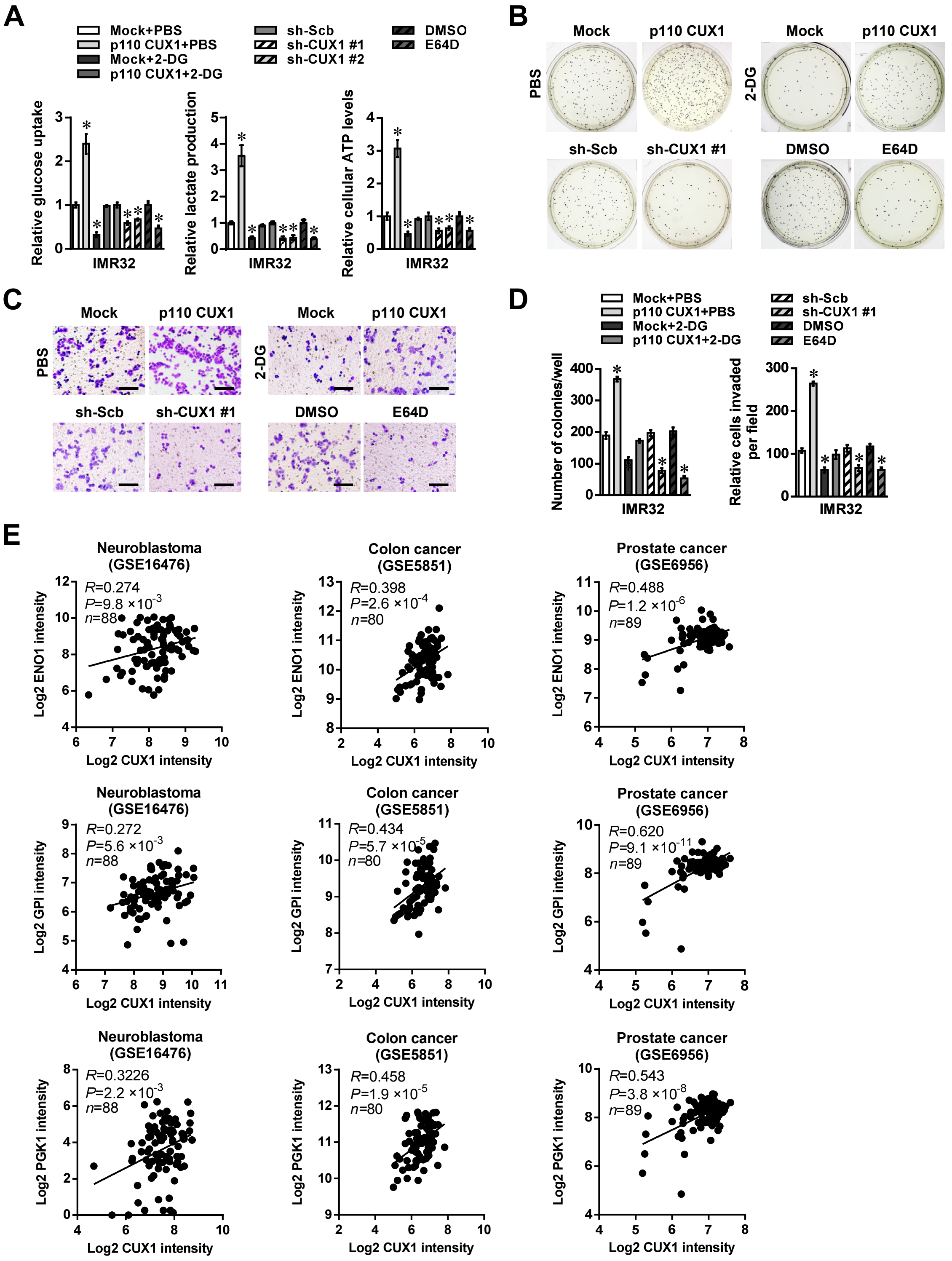

Appendix Figure S2. Roles and expression profiles of *CUX1* and target genes in tumors. **A**, Glucose uptake, lactate production, and ATP levels in IMR32 cells stably transfected with empty vector (mock), *p110 CUX1*, scramble shRNA (sh-Scb), sh-CUX1 #1, or sh-CUX1 #2, and those treated with 2-DG (10 mmol·L^{-1}, for 48rs) or E64D (10 μmol·L^{-1}, for 24 hrs, n=5). Student's t-test, one-way ANOVA, * P<0.05 vs. mock+PBS, sh-Scb, or DMSO. **B-D**, Representative image and quantification (D) of soft agar (B) and matrigel invasion (C) assays showing growth and invasion of IMR32 cells stably transfected with mock, *p110 CUX1,* sh-Scb, or sh-CUX1 #1, and those treated with 2-DG (10 mmol·L^{-1}, for 48rs) or E64D (10 μmol·L^{-1}, for 24 hrs, n=5). Scale bars: 100 μm. Student's t-test, one-way ANOVA, * P<0.05 vs. mock+PBS, sh-Scb, or DMSO. **E**, The expression correlation between *p200 CUX1* and *ENO1*, *GPI*, or *PGK1* in the tissues of NB, colon cancer, and prostate cancer cases derived from public Gene Expression Omnibus (GEO) datasets. Pearson's correlation coefficient analysis. Data information: Data are presented as mean ± SEM. Exact P values are specified in Appendix Table S4.

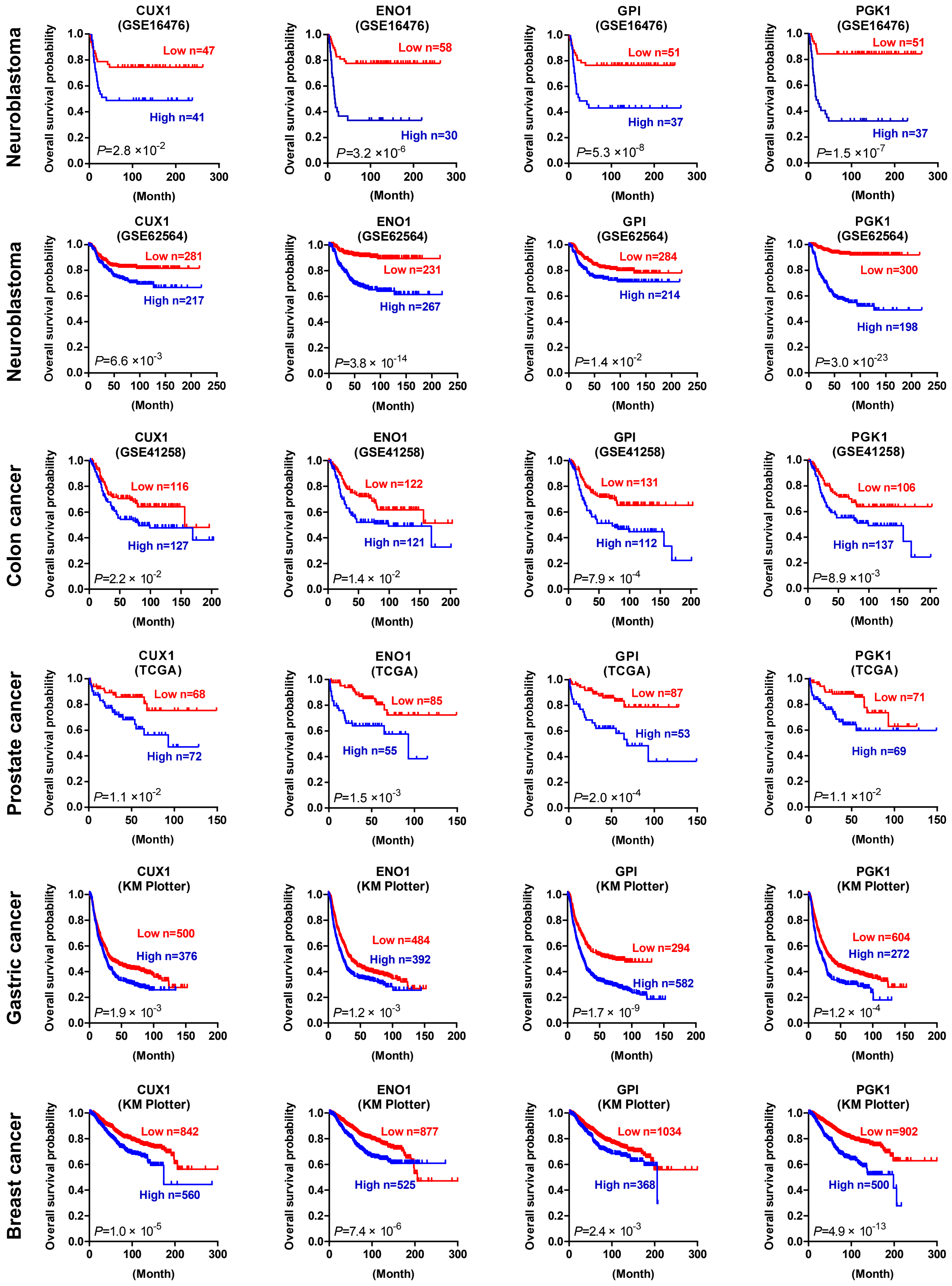

Appendix Figure S3. Expression of *CUX1*, *ENO1*, *GPI*, or *PGK1* is associated with survival of tumor patients. Kaplan–Meier survival curves of *p200 CUX1*, *ENO1*, *GPI*, and *PGK1* in patients with NB, colon cancer, prostate cancer, gastric cancer, or breast cancer derived from GEO datasets, The Cancer Genome Atlas (TCGA), or Kaplan Meier (KM) plotter. Log-rank test for survival comparison.

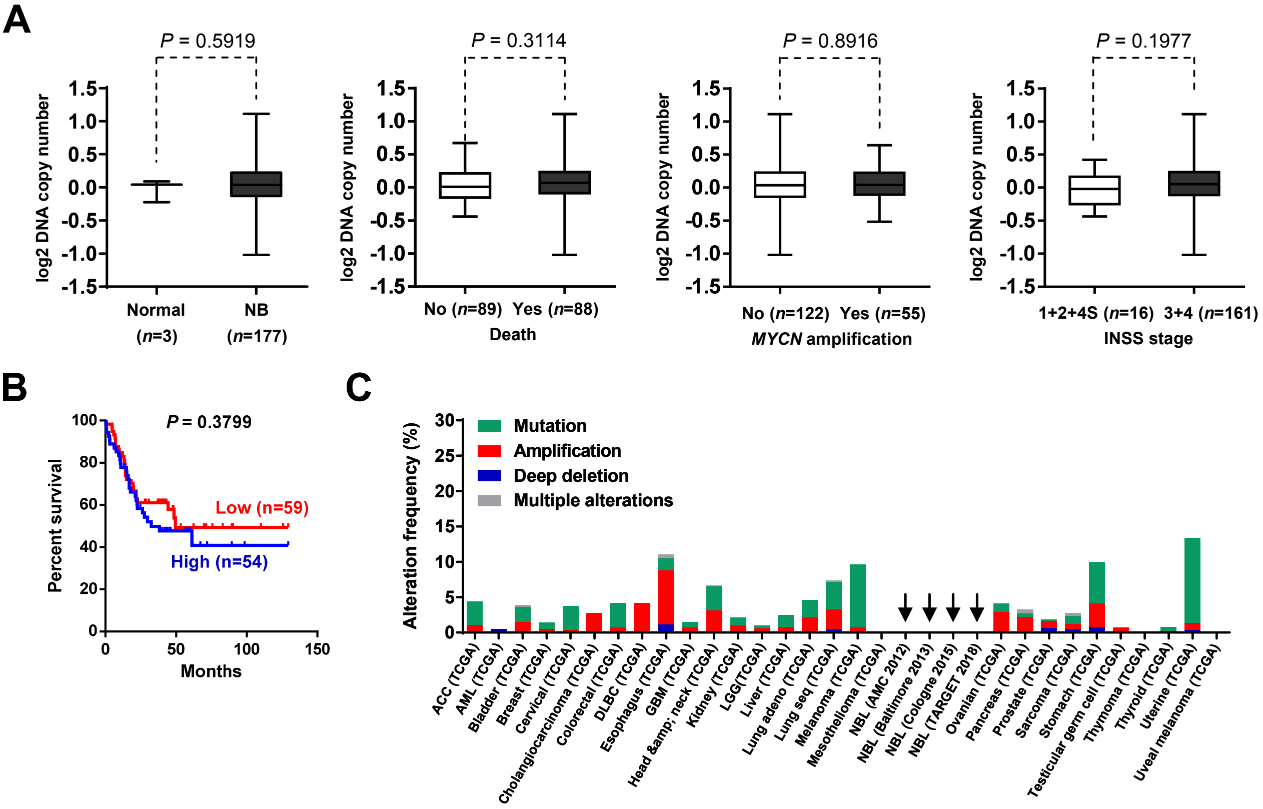

Appendix Figure S4. Copy number and genetic variants of *CUX1* gene in human cancers. A, Mining of Oncogenomics database (https://pob.abcc.ncifcrf.gov/cgi-bin/JK) revealing the copy number of *CUX1* gene locus, locating at chr7: 101460882-101901513, in normal genomic DNA (n=3) and NB tissues (n=177) with varied status of death, *MYCN* amplification, or INSS stages. Bars are means and whiskers (min to max). Student's t-test, P=0.5919, P=0.3114, P=0.8916, and P=0.1977. **B**, Kaplan–Meier survival curves of 113 NB patients with high or low *CUX1* copy number (cutoff value=0.1). Log-rank test, P=0.3799. **C**, Mining of publicly available datasets derived from cBioPortal for Cancer Genomics (http://cbioportal.org) indicating the copy number alteration and genetic variants of *CUX1* in common human cancers, and none of these changes in 563 NB cases of three independent genome-wide studies and tumor alterations relevant for genomics-driven therapy (TARGET, https://software.broadinstitute.org/cancer/cga/target) database (arrowheads).

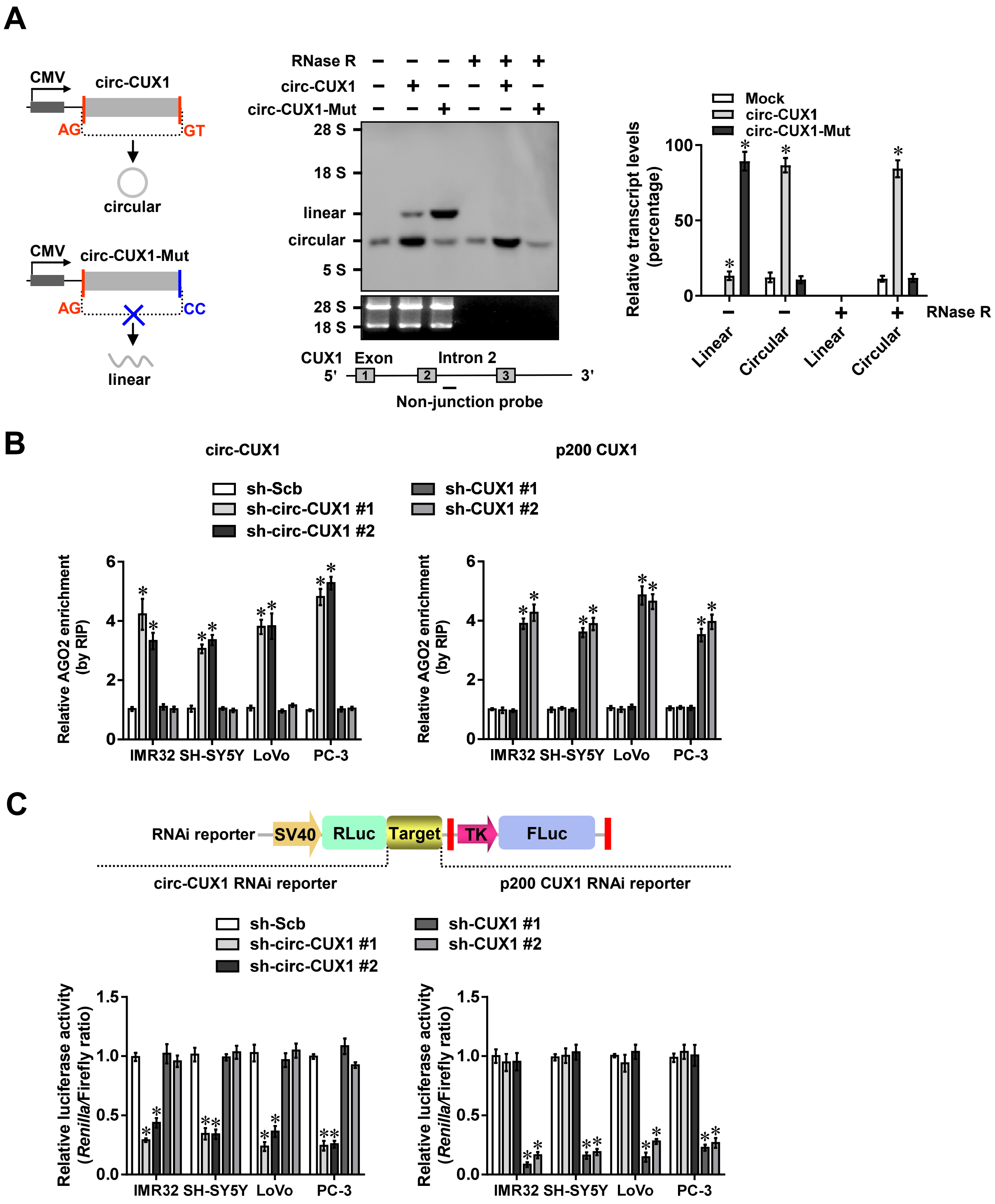

Appendix Figure S5. Specific ectopic expression or knockdown of *circ-CUX1* and *CUX1* in tumor cells. A, Northern blot (middle panel) and quantification (right panel, *n*=6) showing the levels of linear and circular RNA in IMR32 cells transfected with empty vector (mock), *circ-CUX1*, or *circ-CUX1* with mutant back-splicing elements (*circ-CUX1*-Mut, left panel), and those treated with RNase R (3 U·μg^{-1}) digestion. One-way ANOVA, * *P*<0.05 vs. mock. **B**, RIP and real-time qRT-PCR (normalized to input, *n*=6) assays indicating the AGO2 enrichment on *circ-CUX1* or *CUX1* mRNA in tumor cells transfected with scramble shRNA (sh-Scb), sh-circ-CUX1 #1, sh-circ-CUX1 #2, sh-CUX1 #1, or sh-CUX1 #2. One-way ANOVA, * *P*<0.05 vs. sh-Scb. **C**, Dual-luciferase assay showing the activity of *circ-CUX1* or *CUX1* reporter in tumor cells transfected with sh-Scb, sh-circ-CUX1 #1, sh-circ-CUX1 #2, sh-CUX1 #1, or sh-CUX1 #2 (*n*=5). One-way ANOVA, * *P*<0.05 vs. sh-Scb. Data information: Data are presented as mean ± SEM. Exact *P* values are specified in Appendix Table S4.

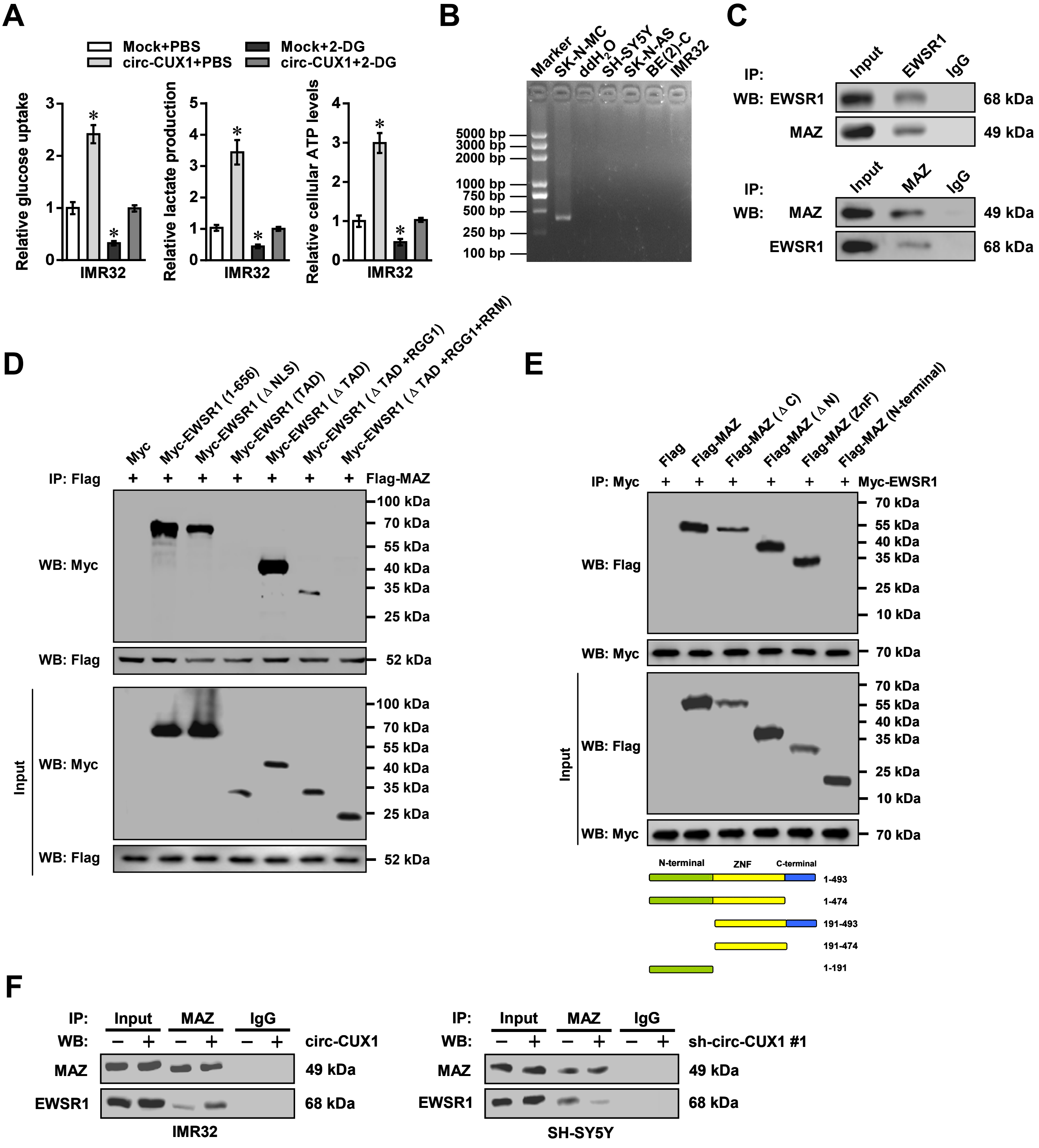

Appendix Figure S6. ***circ-CUX1* regulates aerobic glycolysis and EWSR1-MAZ interaction. A**, Glucose uptake, lactate production, and ATP levels in IMR32 cells stably transfected with empty vector (mock) or *circ-CUX1*, and those treated with 2-DG (10 mmol·L^{-1}) for 48 hrs (n=5). One-way ANOVA, * P<0.05 vs. mock+PBS. **B**, PCR array revealing the detection of *EWSR1-Fli1* fusion gene in SH-SY5Y, SK-N-AS, BE(2)-C, and IMR32 cells, with neuroepithelioma (SK-N-MC) cells and ddH$_2$O$_2$ as controls. **C**, Co-IP and western blot assays indicating the endogenous interaction between EWSR1 and MAZ in IMR32 cells. **D** and **E**, Co-IP and western blot assays (upper panel) revealing the interaction between EWSR1 and MAZ in SH-SY5Y cells transfected with full-length or truncations of Myc-tagged *EWSR1* and FLAG-tagged *MAZ* as indicated (lower panel). **F**, Co-IP and western blot assays indicating the endogenous interaction between EWSR1 and MAZ in IMR32 and SH-SY5Y cells stably transfected with *circ-CUX1* or sh-circ-CUX1 #1. Data information: Data are presented as mean ± SEM. Exact P values are specified in Appendix Table S4.

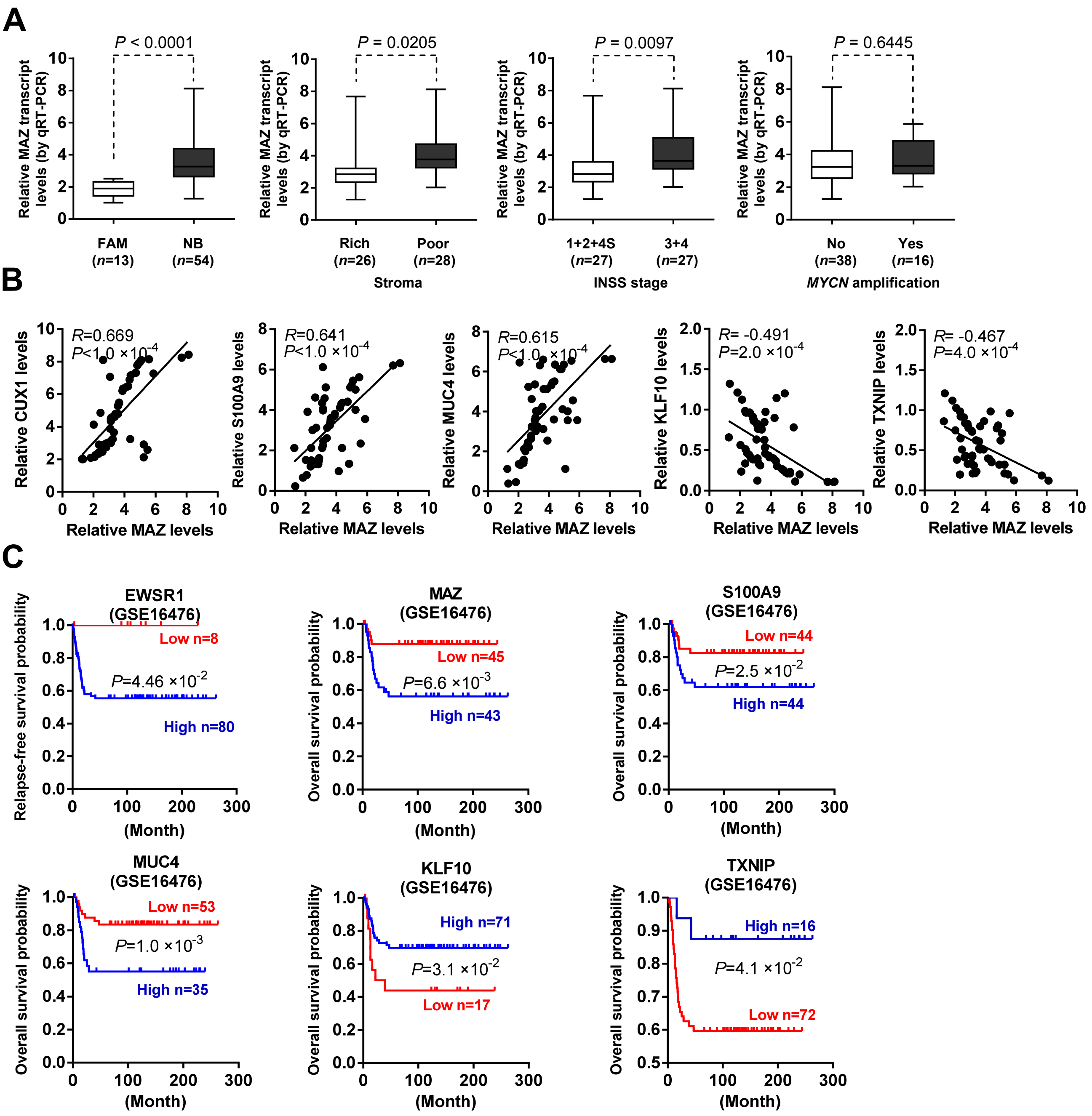

Appendix Figure S7. Expression profiles of MAZ and downstream target genes. A, Real-time qRT-PCR assay indicating the differential levels of *MAZ* (normalized to β-actin) in normal fetal adrenal medulla (FAM, *n*=13) and NB tissues (*n*=54) with different stroma content, INSS stages, or *MYCN*-amplification status. Bars are means and whiskers (min to max). Student's *t*-test. **B**, Real-time qRT-PCR assay showing the expression correlation between *MAZ* and *p200 CUX1*, *S100A9*, *MUC4*, *KLF10*, or *TXNIP* in 54 NB specimens. Pearson's correlation coefficient analysis. **C**, Kaplan–Meier survival plots and log-rank test of 88 NB patients (GSE16476) with low or high expression of *EWSR1* (cutoff value=8.73), *MAZ* (cutoff value=9.31), *S100A9* (cutoff value=6.42), *MUC4* (cutoff value=3.11), *KLF10* (cutoff value=9.89), or *TXNIP* (cutoff value=8.67).

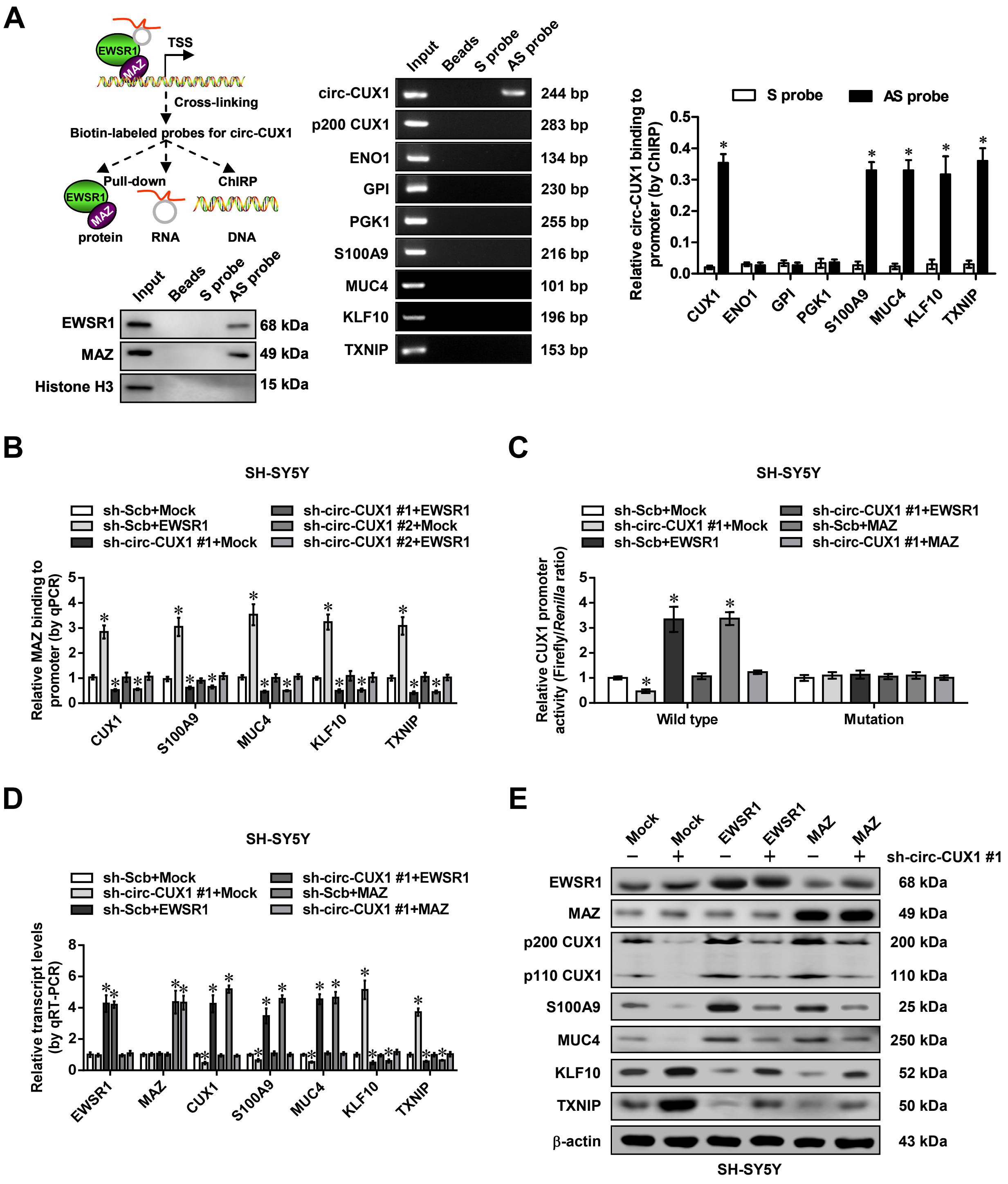

Appendix Figure S8. ***Circ-CUX1* facilitates EWSR1-mediated MAZ transactivation in NB cells. A**, RNA pull-down and ChIRP assays indicating the association of protein (EWSR1 and MAZ, left lower panel), RNA (*circ-CUX1*, *p200 CUX1*, *ENO1*, *GPI*, *PGK1*, *S100A9*, *MUC4*, *KLF10*, or *TXNIP*, middle panel), and target gene promoters (right panel, *n*=5) in SH-SY5Y cells, using biotin-labeled antisense (AS) probe targeting junction site of *circ-CUX1* (left panel). Student's *t*-test, * *P*<0.05 vs. sense (S) probe. **B**, ChIP assay showing the binding of MAZ (normalized to input, *n*=5) to target genes promoters in SH-SY5Y cells stably transfected with scramble shRNA (sh-Scb) or sh-circ-CUX1, and in those co-transfected with *EWSR1*. One-way ANOVA, * *P*<0.05 vs. mock+sh-Scb. **C**, Dual-luciferase assay revealing the relative activity of *p200 CUX1* promoter with wild type or mutant MAZ binding site in SH-SY5Y cells stably transfected with sh-Scb or sh-circ-CUX1 #1, and in those co-transfected with *EWSR1* or *MAZ* (*n*=5). One-way ANOVA, * *P*<0.05 vs. mock+sh-Scb. **D** and **E**, Real-time qPCR (D, normalized to β-actin, *n*=5) and western blot (E) assays showing the expression of *EWSR1*, *MAZ* and their target genes in SH-SY5Y cells stably transfected with sh-Scb or sh-circ-CUX1 #1, and in those co-transfected with *EWSR1* or *MAZ*. One-way ANOVA, * *P*<0.05 vs. mock+sh-Scb. Data information: Data are presented as mean ± SEM. Exact *P* values are specified in Appendix Table S4.

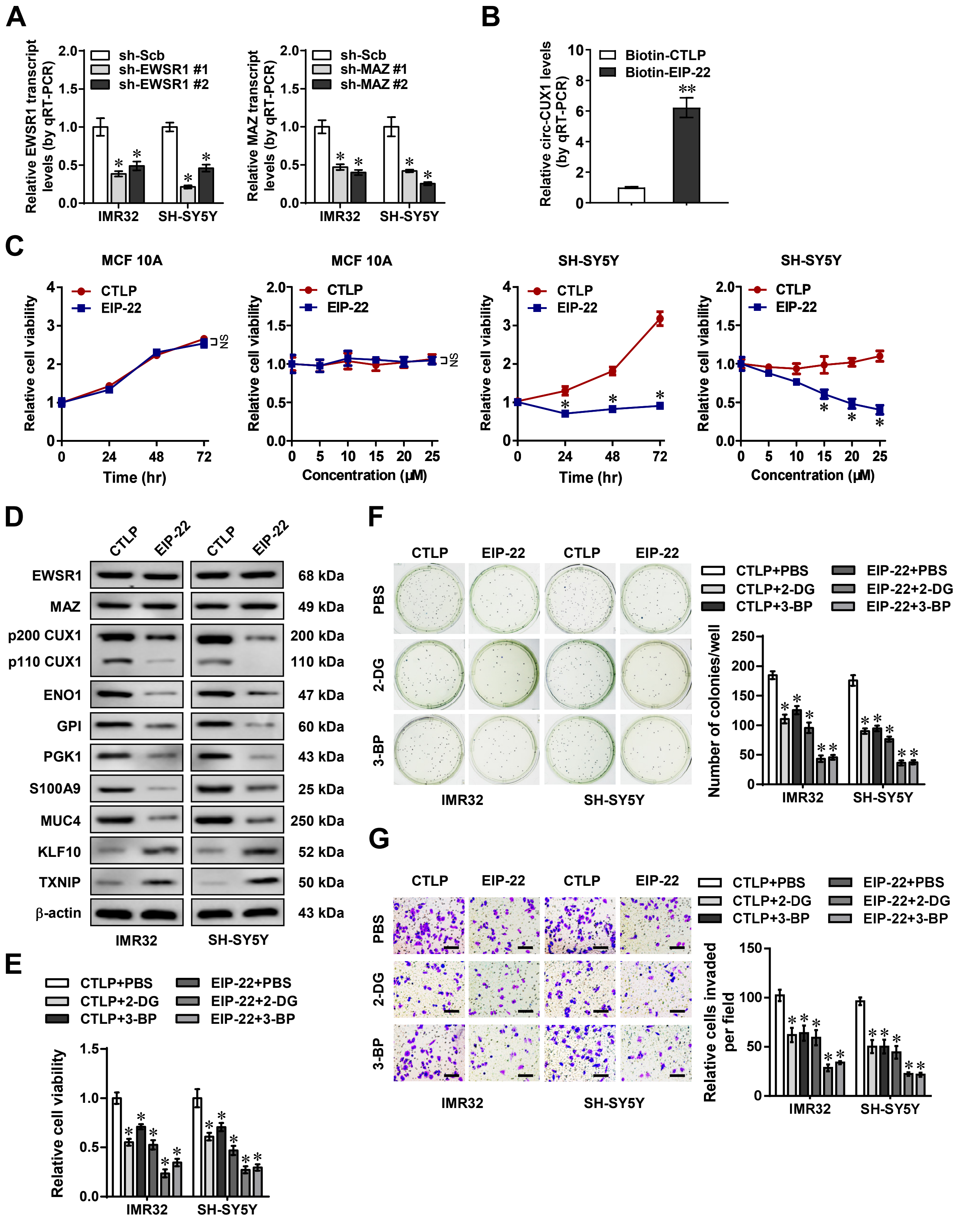

Appendix Figure S9. Gene knockdown and effects of EIP-22 on NB cells *in vitro*. A, Real-time qRT-PCR assay showing the transcript levels of *EWSR1* or *MAZ* in IMR32 and SH-SY5Y cells transfected with scramble shRNA (sh-Scb), sh-EWSR1, or sh-MAZ (*n*=6). One-way ANOVA, * *P*<0.05 vs. sh-Scb. **B**, Peptide pull-down and real-time qRT-PCR assays using biotin-labeled control mutant peptide (CTLP) or EIP-22 indicating their interaction with *circ-CUX1* in SH-SY5Y cells (*n*=5). Student's *t*-test, ** *P*<0.01 vs. CTLP. **C**, MTT assay showing the viability of non-transformed normal MCF 10A and SH-SY5Y cells treated with various concentrations of CTLP or EIP-22 for duration as indicated (*n*=5). One-way ANOVA, Student's *t*-test, * *P*<0.05 vs. CTLP. **D**, Western blot assay revealing the protein levels of EWSR1, MAZ, CUX1, and target genes in IMR32 and SH-SY5Y cells treated with CTLP or EIP-22 (15 μmol·L^{-1}) for 48 hrs. **E**, MTT assay showing the viability of IMR32 and SH-SY5Y cells treated with CTLP, EIP-22 (15 μmol·L^{-1}), 2-DG (10 mmol·L^{-1}), or 3-BP (20 μmol·L^{-1}) for 48 hrs (*n*=5). One-way ANOVA, * *P*<0.05 vs. CTLP+PBS. **F** and **G**, Representative images (left panel) and quantification (right panel) of soft agar (F) and matrigel invasion (G) assays indicating the growth and invasion of IMR32 and SH-SY5Y cells treated with CTLP, EIP-22 (15 μmol·L^{-1}), 2-DG (10 mmol·L^{-1}), or 3-BP (20 μmol·L^{-1}) for 48 hrs (*n*=5). Scale bars: 100 μm. Student's *t*-test, One-way ANOVA, * *P*<0.05 vs. CTLP+PBS. Data information: Data are presented as mean ± SEM. Exact *P* values are specified in Appendix Table S4.

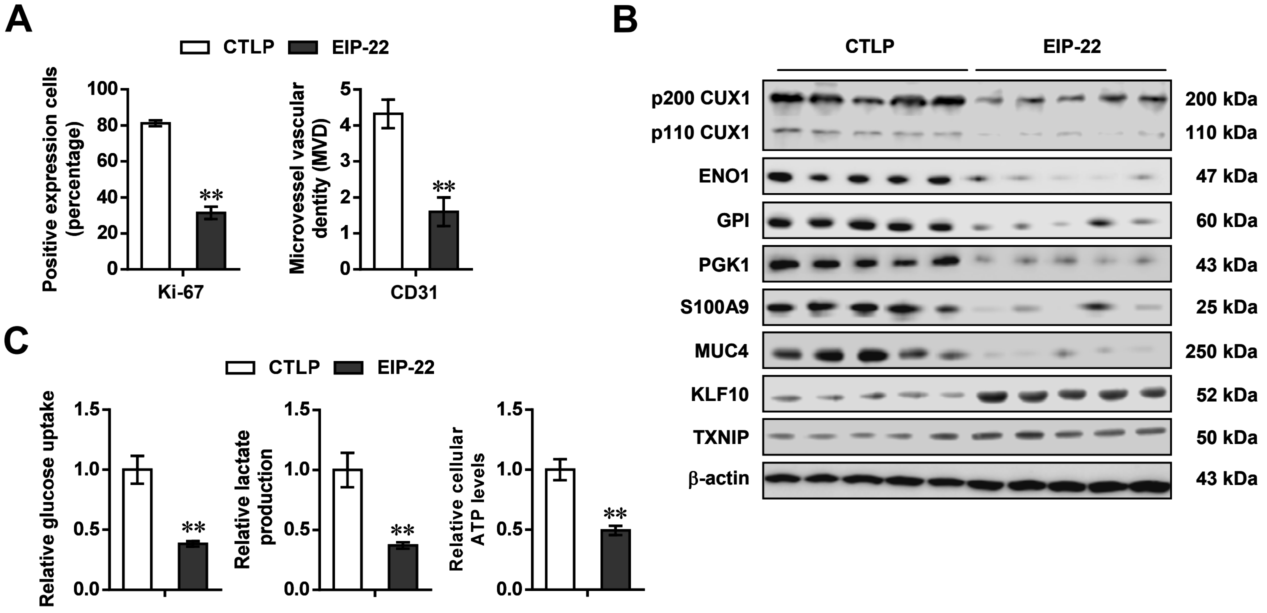

Appendix Figure S10. EIP-22 inhibits tumorigenesis and aerobic glycolysis of NB cells *in vivo*. A, Quantification of immunohistochemical staining showing the expression of Ki-67 and CD31 within subcutaneous xenograft tumors of SH-SY5Y cells following tail vein injection of CTLP or EIP-22 (5 mg·kg^{-1}, *n*=5 for each group). Student's *t*-test, ** *P*<0.01 vs. CTLP. **B**, Western blot assay indicating the expression of *circ-CUX1* target genes in subcutaneous xenograft tumors of SH-SY5Y cells following tail vein injection of CTLP or EIP-22 (5 mg·kg^{-1}, *n*=5 for each group). **C**, Glucose uptake, lactate production, and ATP levels within subcutaneous xenograft tumors of SH-SY5Y cells following tail vein injection of CTLP or EIP-22 (5 mg·kg^{-1}, *n*=5 for each group). Student's *t*-test, ** *P*<0.01 vs. CTLP. Data information: Data are presented as mean ± SEM. Exact *P* values are specified in Appendix Table S4.

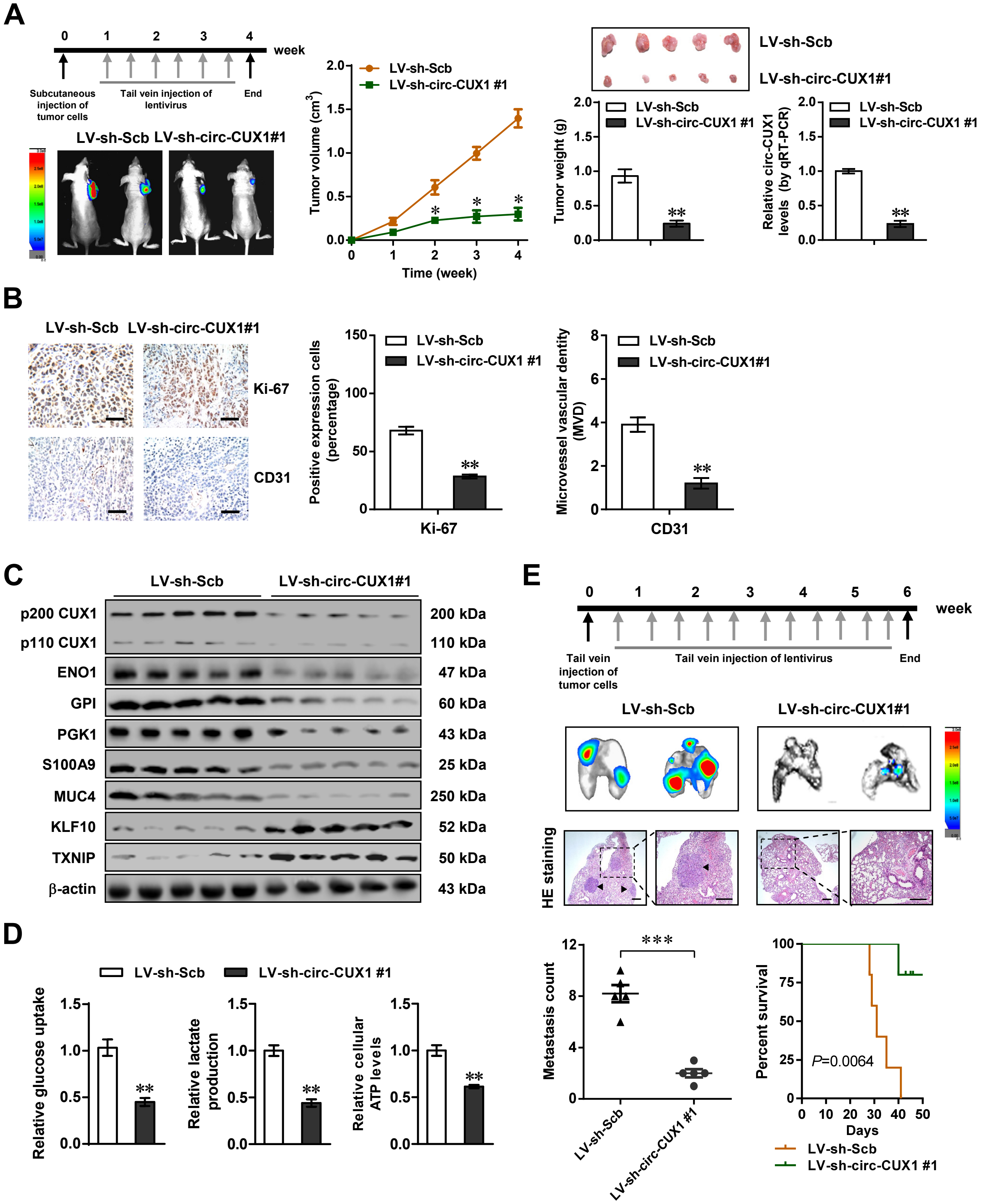

Appendix Figure S11. Lentivirus-mediated *circ-CUX1* knockdown inhibits aerobic glycolysis and tumor progression. A, Representative images, *in vivo* growth curve, weight at the end points, and *circ-CUX1* levels of subcutaneous xenograft tumors formed by IMR32 cells in athymic nude mice (n=5 for each group) that received tail vein injection of lentivirus (LV) carrying scramble shRNA (sh-Scb) or sh-circ-CUX1 #1 as indicated. Student's t-test, one-way ANOVA, * P<0.05, ** P<0.01 vs. LV-sh-Scb. **B**, Representative images and quantification of immunohistochemical staining revealing the expression of Ki-67 and CD31 within subcutaneous xenograft tumors following tail vein injection of lentivirus carrying sh-Scb or sh-circ-CUX1 #1 (n=5 for each group). Scale bars: 100 μm. Student's t-test, ** P<0.01 vs. LV-sh-Scb. **C**, Western blot assay showing the expression of *circ-CUX1* downstream target genes within xenograft tumors (n=5 for each group). **D**, Glucose uptake, lactate production, and ATP levels within subcutaneous xenograft tumors of IMR32 cells following tail vein injection of lentivirus-mediated sh-Scb or sh-circ-CUX1 #1 (n=5 for each group). Student's t-test, ** P<0.01 vs. LV-sh-Scb. **E**, Representative images, HE staining (arrowheads), quantification of lung metastatic colonization, and Kaplan-Meier curves of nude mice (n=5 for each group) treated with tail vein injection of IMR32 cells and lentivirus carrying sh-Scb or sh-circ-CUX1 #1 as indicated (upper panel). Scale bar: 100 μm. Student's t-test, *** P<0.001 vs. LV-sh-Scb. Log-rank test for survival comparison. Data information: Data are presented as mean ± SEM. Exact P values are specified in Appendix Table S4.

Appendix Table S1 Primer sets used for RT-PCR, qRT-PCR, RIP, PCR and ChIP

Primer set	Primers	Sequence	Product size (bp)	Application
circ-CUX1 (divergent)	Forward	5'-CGGCAAACAACCCGTTTCTT-3'	244	RT-PCR, qRT-PCR, RIP
	Reverse	5'-CGCTTTCTGGACTGCTCACT-3'		
hsa_circ_0001732	Forward	5'-AGGTGCTGTTGCTGGAGAAGA-3'	178	RT-PCR
	Reverse	5'-CTGGTGCCTTCTGGATCTGT-3'		
hsa_circ_0001733	Forward	5'-TCACCAAGCTGCGGGAGAATT-3'	130	RT-PCR
	Reverse	5'-GCCAACTCAACTTCTAGGCTG-3'		
hsa_circ_0005253	Forward	5'-CGCTCCAGCCTAGAAGTTGA-3'	220	RT-PCR
	Reverse	5'-TGCCCTCTGGTTTGCCCTTT-3'		
hsa_circ_0006501	Forward	5'-AGCATCCAAGAATTAGTAGC-3'	182	RT-PCR
	Reverse	5'-ACCAGCTCGGTCAGGCTCTT-3'		
hsa_circ_0081606	Forward	5'-AGAAAGCGGCTTATCGAACA-3'	103	RT-PCR
	Reverse	5'-CACTTTCATCCTGCCGGTT-3'		
hsa_circ_0081618	Forward	5'-TGGTGGAGGACGTGCAGAGAC-3'	214	RT-PCR
	Reverse	5'-TCTGGTGCCTTCTGGATCTGT-3'		
hsa_circ_0081619	Forward	5'-ACTCAAAGGCCAGGCTGACT-3'	177	RT-PCR
	Reverse	5'-TCCACCAGCTGTGCGATCTC-3'		
hsa_circ_0081622	Forward	5'-TGGGTCTGAAGGTAACATC-3'	244	RT-PCR
	Reverse	5'-TCAGGCTGGTCTTTCCCTTT-3'		
hsa_circ_0081626	Forward	5'-TGGCTCTGATGAAGCCATCA-3'	125	RT-PCR
	Reverse	5'-CATTTCTTCGTTTCGGTACT-3'		
hsa_circ_0081630	Forward	5'-TGGCATAAGCTCAGTCTGAA-3'	195	RT-PCR
	Reverse	5'-GGTCAGGCTCTTCACCGACT-3'		
hsa_circ_0132814	Forward	5'-CTGCAGGAGACACAGATGTC-3'	250	RT-PCR
	Reverse	5'-TGCTGTCCGAGATCCAAAGC-3'		
hsa_circ_0132815	Forward	5'-CCTACAAACAGCCCTGGAAA-3'	192	RT-PCR
	Reverse	5'-CCTTGTTGTATTCTTCCAGAG-3'		
hsa_circ_0132816	Forward	5'-TCACCTGTCTCAGGACCCCTT-3'	262	RT-PCR
	Reverse	5'-GTGCAGCCGGAGAGAAGAAAT-3'		
hsa_circ_0132817	Forward	5'-CAAGAATTAGTAGCCATGTC-3'	154	RT-PCR
	Reverse	5'-TTCACCGACTCACTGGAACT-3'		
circ-CUX1 (convergent)	Forward	5'-AGTGAGCAGTCCAGAAAGCG-3'	189	qRT-PCR
	Reverse	5'-AAGAAACGGGTTGTTTGCCG-3'		
β-actin (convergent)	Forward	5'-TGCCCATCTACGAGGGGTATG-3'	156	qRT-PCR
	Reverse	5'-TCTCCTTAATGTCACGCACGATTT-3'		
β-actin (divergent)	Forward	5'-AAATCGTGCGTGACATTAAGGAGA-3'	-	qRT-PCR
	Reverse	5'-CATACCCCTCGTAGATGGGCA-3'		
p200 CUX1	Forward	5'-CCAGAGCCTGAACAGACTATTT-3'	283	RT-PCR, qRT-PCR
	Reverse	5'-CTTTAAGGCAGGGTCGAGGGCA-3'		
p75 CUX1	Forward	5'-GCTATTTTCAGGCACGGTTTCTC-3'	366	RT-PCR, qRT-PCR
	Reverse	5'-GGTCTCCCCAAATAAGCGCTGG-3'		
CASP	Forward	5'-GGAGGTGCTGTTGCTGGAGAAG-3'	196	RT-PCR, qRT-PCR
	Reverse	5'-CCGCTGGATGGACTGAATGATGCT-3'		
ALDOA	Forward	5'-GTGGGCATCAAGGTAGACAA-3'	226	qRT-PCR
	Reverse	5'-GCTGGCAGATACTGGCATAA-3'		
ENO1	Forward	5'-GATGACTGGGGAGCTTGGCAGAAG-3'	134	qRT-PCR
	Reverse	5'-TTGAGCAGGAGGCAGTTGCAGGAC-3'		
GAPDH	Forward	5'-GGGCTGCTTTTAACTCTGGT-3'	198	qRT-PCR
	Reverse	5'-TGATTTTGGAGGGATCTCGC-3'		
GPI	Forward	5'-AGTTCTGGGATTGGGTGGGA-3'	230	qRT-PCR
	Reverse	5'-ATAGGGCAGCATGGCGTGTG-3'		
HK2	Forward	5'-TGGAGCGAGGTCTGAGCAAG-3'	145	qRT-PCR
	Reverse	5'-ACCAGCAGGACCCGGAAATT-3'		
LDHA	Forward	5'-TGTCTCTGGCAAAGTGGAT-3'	117	qRT-PCR
	Reverse	5'-ATTAGGTAACGGAATCGGG-3'		
PGK1	Forward	5’-AGCCAAGATTGTCAAAGACCT-3'	255	qRT-PCR
	Reverse	5’-GCTTCCCATTCAAATACCCC-3'		
PKM	Forward	5’-ACGGAGGTGGAAAATGGTGG-3'	162	qRT-PCR
	Reverse	5’-TGCCTTGCGGATGAATGACG-3'		
MAZ	Forward	5'-CTGCCTTGGAGAAGAAGACA-3'	142	qRT-PCR
	Reverse	5'-ATAGCACCCGAGGGGACCCG-3'		
S100A9	Forward	5'-TCATCAACACCTTCCACCAA-3'	216	qRT-PCR
	Reverse	5'-TTAGCCTCGCCATCAGCA-3'		
MUC4	Forward	5'-CAGGCCACCAACTTCATCG-3'	101	qRT-PCR
	Reverse	5'-ACACGGATTGCGTCGTGAG-3'		
KLF10	Forward	5'-GAGTCACATCTGTAGCCACCC-3'	196	qRT-PCR
	Reverse	5'-GGGGCACGCAAATTTCTTCTC-3'		
TXNIP	Forward	5'-CAGACTTCGGAGTACCTGCGCTAT-3'	153	qRT-PCR
	Reverse	5'-TTTGAAGGATGTTCCCAGAGGC-3'		
U1	Forward	5'-ACTTACCTGGCAGGGGAGATACC-3'	137	qRT-PCR
	Reverse	5'-CCACTACCACAAATTATGCAGTCG-3'		
pri-miR-222	Forward	5'-TGCCCAATAATCTCTCTCAGGA-3'	170	qRT-PCR
	Reverse	5'-CTCCCCAGAAGGCAAAGGAT-3'		
circACC1	Forward	5'-TGAAATGTTTCGAAATGA-3'	131	qRT-PCR
	Reverse	5'-TCATTATCTTCAACCCTCTAG-3'		
EWSR1-FLI1	Forward	5'-CCCACTAGTTACCCACCCCAA-3'	355	PCR
	Reverse	5'-ACTCCCCGTTGGTCCCCTCC-3'		
CUX1 (-1857/-1713)	Forward	5'-GCCACCGCAGAAATAGACATG-3'	145	ChIP
	Reverse	5'-CTGGCGGGAAGCGGCATT-3'		
ENO1 (-1690/-1461)	Forward	5'-ATTCACTCTCCCTTGATCCCGG-3'	236	ChIP
	Reverse	5'-TCCATAGCTCCTTAGGCTTTGT-3'		
GPI (-1358/-1201)	Forward	5'-GGGTAAGTGATAATGGGGCA-3'	158	ChIP
	Reverse	5'-TCTACGGGGGTTCTGAGAAA-3'		
PGK1 (-621/-358)	Forward	5'-GCGTTTTGTGAGCGTTGGTT-3'	264	ChIP
	Reverse	5'-ATTCGAGGCTGCCCTACCAG-3'		
S100A9 (-160/+28)	Forward	5'-CAGAAGGCGCTCTGTGAA-3'	188	ChIP
	Reverse	5'-CAAAGCCGAGGAGCCACA-3'		
MUC4 (-223/-115)	Forward	5'-TGGCCCACCCATTTCCAG-3'	109	ChIP
	Reverse	5'-CACCGATACACCCGTTGCT-3'		
KLF10 (-297/-154)	Forward	5'-GGACGAGGGGCAAGGAGTG-3'	144	ChIP
	Reverse	5'-CGTGAAGCTGGTGTCAGTGGA-3'		
TXNIP (-866/-731)	Forward	5'-TTCCCCAACTTTGCAGGTA-3'	136	ChIP
	Reverse	5'-GCTGTTCGTCACCCACCC-3'		

circ-CUX1, circular RNA generated from CUX1; CUX1, CUT-like homeobox 1; CASP, CDP/cut alternatively spliced cDNA; ALDOA, aldolase, fructose-bisphosphate A; ENO1, enolase 1; GAPDH, glyceraldehyde 3-phosphate dehydrogenase; GPI, glucose-6-phosphate isomerase; HK2, hexokinase 2; LDHA, lactate dehydrogenase A; PGK1, phosphoglycerate kinase 1; PKM, pyruvate kinase M; MAZ, MYC associated zinc finger protein; S100A9, S100 calcium binding protein A9; MUC4, mucin 4; KLF10, Kruppel like factor 10; TXNIP, thioredoxin interacting protein; pri-miR-222, primary microRNA 222; circACC1, circular RNA generated from CUX1; EWSR1, EWS RNA binding protein 1.

Appendix Table S2 Oligonucleotide sets used for constructs, guide DNA, and probes

Oligo Set	Sequences
pLCDH-circ-CUX1	5'-CCGGAATTCTGAAATATGCTATCTTACAGAGAGAACTCGATGCCACCGCAAC-3' (sense);<br>5'-CGCGGATCCTCAAGAAAAAATATATTCACCTGCCCACGTGTTCTTACAGACC-3' (antisense)
pLCDH-circ-CUX1-Mut	5'-ACACGTGGGCAGCCGAATATATTTTTTCTTGAGGATCCAC-3' (sense);<br>5'-ATATTCGGCTGCCCACGTGTTCTTACAGACCAAGCTTGTC-3' (antisense)
pGL3-ENO1 (-1880/+301)	5'-CGGGGTACCAGCTACATCCCCTTCTGCACTCATC-3' (sense);<br>5'-GCCGCTCGAGGCCGTCACTCATTCCCTCACCTCCC-3' (antisense)
pGL3-GPI (-1854/+247)	5'-CGGGGTACCTCTCTGATGGATTCAGGCAGGGGTA-3' (sense);<br>5'-GCCGCTCGAGGAAGTGGCAGTGGGGAGGGTGGGCA-3' (antisense)
pGL3-PGK1 (-882/+246)	5'-CGGGGTACCCTAAGAACTTGGACACCCTCCACG-3' (sense);<br>5'-GCCGCTCGAGCAGAATTACCTCATAACGACCCGC-3' (antisense)
pGL3-CUX1 (-2084/+106)	5'-CGGGGTACCAACCAGATGTATTTCGGGAGTA-3' (sense);<br>5'-CGGAAGATCTGTGAGCGGCTGATAGAGAGGGG-3' (antisense)
pGL3-CUX1 (ΔMAZ)	5'-GCACCCCCCCATTAACCCCCGCCGCCTCCCCGAGTGTGCAGGCAT-3' (sense);<br>5'-GCGGCGGGGGTTAATGGGGGGGTGCTTCCGCCTTCCCCTGGCATG-3' (antisense)
psiCHECK-circ-CUX1 reporter	5'-GCCGCTCGAGCGGCAAACAACCCGTTTCTT-3' (sense);<br>5'-ATTTGCGGCCGCCGCTTTCTGGACTGCTCACT-3' (antisense)
psiCHECK-CUX1 reporter	5'-GCCGCTCGAGATGGCGGCCAATGTGGGATCGAT-3' (sense);<br>5'-ATTTGCGGCCGCCAGTTTTCTGCACTTGGAGCTCC-3' (antisense)
pGL3-CUX1-reporter	5'-CTATATCGATTATCTCATCGATCATCTTATCAATTATCTCATCGATTATA-3' (sense);<br>5'-AGCTTATAATCGATGAGATAATTGATAAGATGATCGATGAGATAATCGATATAGGTAC-3' (antisense)
pGL3-MAZ-reporter	5'-CGGGAGGGGATGGGCGGGGCGGGGAGGGGCTGGGCGGGGAGGGGGAGGGA-3' (sense);<br>5'- AGCTTCCCTCCCCCTCCCCGCCCAGCCCCTCCCCGCCCCGCCCATCCCCTCCCGGTAC-3' (antisense)
pcDNA3.1-circ-CUX1	5'-CGCGGATCCAGAGAACTCGATGCCACCGCAACG-3' (sense);<br>5'-GCCGCTCGAGCTGCCCACGTGTTCTTACAGACCA-3' (antisense)
pBiFC-VN173-EWSR1	5'-CCCAAGCTTATGGCGTCCACGGATTACAGT-3' (sense)<br>5'-CCGGAATTCCCGTAGGGCCGATCTCTGCGCTC-3' (antisense)
pBiFC-VC155-MAZ	5'-CCGGAATTCGGATGTTCCCGGTGTTTCCTTGC-3' (sense);<br>5'-GCCGCTCGAGCGCAGGTGGGCTGTGGCTGGGG-3' (antisense)
pCMV-N-Myc-EWSR1	5'-CCGGAATTCATGGCGTCCACGGATTACAGTAC-3' (sense);<br>5'-GCCGCTCGAGCTAGTAGGGCCGATCTCTGCGCT-3' (antisense)
pCMV-N-Myc-EWSR1 (Δ NLS)	5'-CCGGAATTCATGGCGTCCACGGATTACAGTAC-3' (sense);<br>5'-GCCGCTCGAGTCCTCCACGTCCTCCTCTTCTTC-3' (antisense)
pCMV-N-Myc-EWSR1 (TAD)	5'-CCGGAATTCATGGCGTCCACGGATTACAGTAC-3' (sense);<br>5'-GCCGCTCGAGTTGGCTATATTGACTTGGAGCTT-3' (antisense)
pCMV-N-Myc-EWSR1 (ΔTAD)	5'-CCGGAATTCCAGAGCAGCAGCTACGGGCAGCA-3' (sense);<br>5'-GCCGCTCGAGCTAGTAGGGCCGATCTCTGCGCT-3' (antisense)
pCMV-N-Myc-EWSR1 (ΔTAD+RGG1)	5'-CCGGAATTCAGTGCAATTTATGTACAAGGATT-3' (sense);<br>5'-GCCGCTCGAGCTAGTAGGGCCGATCTCTGCGCT-3' (antisense)
pCMV-N-Myc-EWSR1 (ΔTAD+RGG1+RRM)	5'-CCGGAATTCAAGCCTCCAATGAACAGTATGCG-3' (sense);<br>5'-GCCGCTCGAGCTAGTAGGGCCGATCTCTGCGCT-3' (antisense)
pCMV-3Flag-p110 CXU1	5'-CCGGAATTCTCCACCTCGCCCATGCCCACCGT-3' (sense);<br>5'-GCCGCTCGAGTCAGAACTCCCATTCGATAGGTT-3' (antisense)
pCMV-3Flag-MAZ	5'-CGCGGATCCATGTTCCCGGTGTTTCCTTGC-3' (sense);<br>5'-GCCGCTCGAGTCAGCAGGTGGGCTGTGGCTG-3' (antisense)
pCMV-3Flag-MAZ (ΔC)	5'-CGCGGATCCATGTTCCCGGTGTTTCCTTGC-3' (sense);<br>5'-GCCGCTCGAGATGGGTCTGCATGTGGCCGGC-3' (antisense)
pCMV-3Flag-MAZ (ΔN)	5'-CGCGGATCCTGCGCTCTGTGCGCCAAGGAG-3' (sense);<br>5'-GCCGCTCGAGTCAGCAGGTGGGCTGTGGCTG-3' (antisense)
pCMV-3Flag-MAZ (ZNF)	5'-CGCGGATCCTGCGCTCTGTGCGCCAAGGAG-3' (sense);<br>5'-GCCGCTCGAGATGGGTCTGCATGTGGCCGGC-3' (antisense)
pCMV-3Flag-MAZ (N-terminal)	5'-CGCGGATCCATGTTCCCGGTGTTTCCTTGC-3' (sense);<br>5'-GCCGCTCGAGGATGTAGGGCCCCTTGCTCTT-3' (antisense)
pCMV-EWSR1 Mut-1 (RRM domain)	5'-CAAGAGAACTTGGCAATGCATGATCCACATCTACCTGGACAAGGAAACA-3' (sense);<br>5'-TGTGGATCATGCATTGCCAAGTTCTCTTGTTCATCTTAACAACCCCACA-3' (antisense)
pCMV-EWSR1 Mut-2 (RRM domain)	5'-CAAGGAAACATGCATGCCCGAAGGCGATGCCACAGTGTCCTATGAAGACCC-3' (sense);<br>5'-GGCATCGCCTTCGGGCATGCATGTTTCCTTGTCCAGGTAGATGTGGATCAT-3' (antisense)
Guide Oligo	5'-AGCCAGCTTGGGTCTAGCTGCCCACGTGTT-3'
circ-CUX1 non-junction probe	5'-GCTGGAAATGGGGCTGAAGACATCTG-3'
circ-CUX1 antisense probe	5'-GGTGGCATCGAGTTCTCTCTGCCCACGTGTTCTTAC-3'
circ-CUX1 sense probe	5'-GTAAGAACACGTGGGCAGAGAGAACTCGATGCCACC-3'
p200 CUX1 antisense probe	5'-TCATCTTGTGAAATGGCTCCTTGCCACGAA-3'
CASP antisense probe	5'-ATCTTCTCAAAGAGCTTGATGTTGTCGGCG-3'
pri-miR-222 antisense probe	5'-GGCAAAGGATCACCCAGCTGCTGGAAGGTGTAGGTACCCTCAATGGCTCAGTAGCCAGTGTAGATCCTGTCTTT CGTA-3'
circACC1 antisense probe	5'-TATTTCAAAGTCTGCATTGGC-3'

circ-CUX1, circular RNA generated from CUX1; CUX1, CUT-like homeobox 1; ENO1, enolase 1; GPI, glucose-6-phosphate isomerase; PGK1,phosphoglycerate kinase 1; EWSR1, EWS RNA binding protein 1; MAZ, MYC associated zinc finger protein; CASP, CDP/cut alternatively spliced cDNA; pri-miR-222, primary microRNA 222; circACC1, circular RNA generated from CUX1.

Appendix Table S3 Oligonucleotides encoding short hairpin RNAs

Oligo Set	Sequences	
sh-Scb	5'-CCGGGCGAACGATCGAGTAAACGGACTCGAGTCCGTTTACTCGATCGTTCGCTTTTT-3'	(sense);
	5'-AATTCAAAAAGCGAACGATCGAGTAAACGGACTCGAGTCCGTTTACTCGATCGTTCGC-3'	(antisense)
sh-CUX1 #1	5'-CCGGGCGGCAAGGAGCCATTTCACACTCGAGTGTGAAATGGCTCCTTGCCGCTTTTTG-3'	(sense);
	5'-AATTCAAAAAGCGGCAAGGAGCCATTTCACACTCGAGTGTGAAATGGCTCCTTGCCGC-3'	(antisense)
sh-CUX1 #2	5'-CCGGGCGGAGACTGGCTCTGATGAACTCGAGTTCATCAGAGCCAGTCTCCGCTTTTTG-3'	(sense);
	5'-AATTCAAAAAGCGGAGACTGGCTCTGATGAACTCGAGTTCATCAGAGCCAGTCTCCGC-3'	(antisense)
sh-CASP #1	5'-CCGGTCGACAACATCAAGCTCTTTCTCGAGAAAGAGCTTGATGTTGTCGTTTTTG-3'	(sense);
	5'-GATCCAAAAACGACAACATCAAGCTCTTTCTCGAGAAAGAGCTTGATGTTGTCGA-3'	(antisense)
sh-CASP #2	5'-CCGGTGCTTCTTCTACACACTGTTCTCGAGAACAGTGTGTAGAAGAAGCTTTTTG-3'	(sense);
	5'-GATCCAAAAAGCTTCTTCTACACACTGTTCTCGAGAACAGTGTGTAGAAGAAGCA-3'	(antisense)
sh-hsa_circ_0132817	5'-CCGGTACAACCTCGAAAGCACTCCCTCGAGGGAGTGCTTTCGAGGTTGTTTTTTG-3'	(sense);
	5'-GATCCAAAAAACAACCTCGAAAGCACTCCCTCGAGGGAGTGCTTTCGAGGTTGTA-3'	(antisense)
sh-hsa_circ_0005253	5'-CCGGTGCACACTCAAAGGCCGACGCTCGAGCGTCGGCCTTTGAGTGTGCTTTTTG-3'	(sense);
	5'-GATCCAAAAAGCACACTCAAAGGCCGACGCTCGAGCGTCGGCCTTTGAGTGTGCA-3'	(antisense)
sh-circ-CUX1 #1	5'-CCGGAAGAACACGTGGGCAGAGAGACTCGAGTCTCTCTGCCCACGTGTTCTTTTTTTG-3'	(sense);
	5'-GATCCAAAAAAAGAACACGTGGGCAGAGAGACTCGAGTCTCTCTGCCCACGTGTTCTT-3'	(antisense)
sh-circ-CUX1 #2	5'-CCGGGAACACGTGGGCAGAGAGAACCTCGAGGTTCTCTCTGCCCACGTGTTCTTTTTG-3'	(sense);
	5'-GATCCAAAAAGAACACGTGGGCAGAGAGAACCTCGAGGTTCTCTCTGCCCACGTGTTC-3'	(antisense)
sh-EWSR1 #1	5'-CCGGGCCCAGCCCACTCAAGGATATCTCGAGATATCCTTGAGTGGGCTGGGCTTTTTG-3'	(sense);
	5'-GATCCAAAAAGCCCAGCCCACTCAAGGATATCTCGAGATATCCTTGATGGGGCTGGGC-3'	(antisense)
sh-EWSR1 #2	5'-CCGGCAGCAGAGTAGCTATGGTCAACTCGAGTTGACCATAGCTACTCTGCTG TTTTTG-3'	(sense);
	5'-GATCCAAAAACAGCAGAGTAGCTATGGTCAACTCGAGTTGACCATAGCTACTCTGCTG-3'	(antisense)
sh-MAZ #1	5'-CCGGCGGCTTATATTTCGGACCACACTCGAGTGTGGTCCGAAATATAAGCCGTTTTTG-3'	(sense);
	5'-AATTCAAAAACGGCTTATATTTCGGACCACACTCGAGTGTGGTCCGAAATATAAGCCG-3'	(antisense)
sh-MAZ #2	5'-CCGGCGGCCCTTCAAATGTGAGAAACTCGAGTTTCTCACATTTGAAGGGCCGTTTTTG-3'	(sense);
	5'-AATTCAAAAACGGCCCTTCAAATGTGAGAAACTCGAGTTTCTCACATTTGAAGGGCCG-3'	(antisense)

CUX1, CUT-like homeobox 1; CASP, CDP/cut alternatively spliced cDNA; circ-CUX1, circular RNA generated from CUX1; EWSR1, EWS RNA binding protein 1; MAZ, MYC associated zinc finger protein.

Appendix Table S4 *P* values for differences between experimental groups in figures

Figure	Comparison	Sample number	*P*-value	Statistical analysis
Figure 1B	CUX1: CUX1 vs. Mock	5	0.0119	Student's unpaired *t*-test
	CUX1: sh-CUX1 #1 vs. sh-Scb	5	0.0114	One-way ANOVA
	CUX1: sh-CUX1 #2 vs. sh-Scb	5	0.0142	
	ALDOA: CUX1 vs. Mock	5	0.4745	Student's unpaired *t*-test
	ALDOA: sh-CUX1 #1 vs. sh-Scb	5	0.7946	One-way ANOVA
	ALDOA: sh-CUX1 #2 vs. sh-Scb	5	0.7997	
	ENO1: CUX1 vs. Mock	5	0.0113	Student's unpaired *t*-test
	ENO1: sh-CUX1 #1 vs. sh-Scb	5	0.0117	One-way ANOVA
	ENO1: sh-CUX1 #2 vs. sh-Scb	5	0.0146	
	GAPDH: CUX1 vs. Mock	5	0.7163	Student's unpaired *t*-test
	GAPDH: sh-CUX1 #1 vs. sh-Scb	5	0.9839	One-way ANOVA
	GAPDH: sh-CUX1 #2 vs. sh-Scb	5	0.9561	
	GPI: CUX1 vs. Mock	5	0.0102	Student's unpaired *t*-test
	GPI: sh-CUX1 #1 vs. sh-Scb	5	0.0223	One-way ANOVA
	GPI: sh-CUX1 #2 vs. sh-Scb	5	0.0297	
	HK2: CUX1 vs. Mock	5	0.9857	Student's unpaired *t*-test
	HK2: sh-CUX1 #1 vs. sh-Scb	5	0.559	One-way ANOVA
	HK2: sh-CUX1 #2 vs. sh-Scb	5	0.5847	
	LDHA: CUX1 vs. Mock	5	0.7032	Student's unpaired *t*-test
	LDHA: sh-CUX1 #1 vs. sh-Scb	5	0.6017	One-way ANOVA
	LDHA: sh-CUX1 #2 vs. sh-Scb	5	0.7074	
	PGK1: CUX1 vs. Mock	5	0.0103	Student's unpaired *t*-test
	PGK1: sh-CUX1 #1 vs. sh-Scb	5	0.0429	One-way ANOVA
	PGK1: sh-CUX1 #2 vs. sh-Scb	5	0.0492	
	PKM: CUX1 vs. Mock	5	0.8555	Student's unpaired *t*-test
	PKM: sh-CUX1 #1 vs. sh-Scb	5	0.7341	One-way ANOVA
	PKM: sh-CUX1 #2 vs. sh-Scb	5	0.8535	
Figure 1D	ENO1: p110 CUX1 vs. Mock ; IMR32	5	0.0104	Student's unpaired *t*-test
	ENO1: sh-CUX1 #1 vs. sh-Scb; IMR32	5	0.0113	One-way ANOVA
	ENO1: sh-CUX1 #2 vs. sh-Scb; IMR32	5	0.0211	
	ENO1: E64D vs. DMSO; IMR32	5	0.0123	Student's unpaired *t*-test
	GPI: CUX1 vs. Mock ; IMR32	5	0.0109	Student's unpaired *t*-test
	GPI: sh-CUX1 #1 vs. sh-Scb; IMR32	5	0.0112	One-way ANOVA
	GPI: sh-CUX1 #2 vs. sh-Scb; IMR32	5	0.0268	
	GPI: E64D vs. DMSO; IMR32	5	0.0106	Student's unpaired *t*-test
	PGK1: CUX1 vs. Mock ; IMR32	5	0.0123	Student's unpaired *t*-test
	PGK1: sh-CUX1 #1 vs. sh-Scb; IMR32	5	0.0244	One-way ANOVA
	PGK1: sh-CUX1 #2 vs. sh-Scb; IMR32	5	0.0194	
	PGK1: E64D vs. DMSO; IMR32	5	0.0134	Student's unpaired *t*-test
	ENO1: CUX1 vs. Mock ; SH-SY5Y	5	0.0108	Student's unpaired *t*-test
	ENO1: sh-CUX1 #1 vs. sh-Scb; SH-SY5Y	5	0.0183	One-way ANOVA
	ENO1: sh-CUX1 #2 vs. sh-Scb; SH-SY5Y	5	0.033	
	ENO1: E64D vs. DMSO; SH-SY5Y	5	0.0135	Student's unpaired *t*-test
	GPI: CUX1 vs. Mock ; SH-SY5Y	5	0.0108	Student's unpaired *t*-test
	GPI: sh-CUX1 #1 vs. sh-Scb; SH-SY5Y	5	0.0159	One-way ANOVA
	GPI: sh-CUX1 #2 vs. sh-Scb; SH-SY5Y	5	0.0151	
	GPI: E64D vs. DMSO; SH-SY5Y	5	0.0207	Student's unpaired *t*-test
	PGK1: CUX1 vs. Mock ; SH-SY5Y	5	0.0102	Student's unpaired *t*-test
	PGK1: sh-CUX1 #1 vs. sh-Scb; SH-SY5Y	5	0.0111	One-way ANOVA
	PGK1: sh-CUX1 #2 vs. sh-Scb; SH-SY5Y	5	0.0221	
	PGK1: E64D vs. DMSO; SH-SY5Y	5	0.0129	Student's unpaired *t*-test
Figure 1E	ENO1: CUX1 vs. Mock ; IMR32	5	0.0101	Student's unpaired *t*-test
	ENO1: sh-CUX1 #1 vs. sh-Scb; IMR32	5	0.0123	One-way ANOVA
	ENO1: sh-CUX1 #2 vs. sh-Scb; IMR32	5	0.0137	
	ENO1: E64D vs. DMSO; IMR32	5	0.0221	Student's unpaired *t*-test
	GPI: CUX1 vs. Mock ; IMR32	5	0.0206	Student's unpaired *t*-test
	GPI: sh-CUX1 #1 vs. sh-Scb; IMR32	5	0.0157	One-way ANOVA
	GPI: sh-CUX1 #2 vs. sh-Scb; IMR32	5	0.0277	
	GPI: E64D vs. DMSO; IMR32	5	0.0108	Student's unpaired *t*-test
	PGK1: CUX1 vs. Mock ; IMR32	5	0.0102	Student's unpaired *t*-test
	PGK1: sh-CUX1 #1 vs. sh-Scb; IMR32	5	0.0106	One-way ANOVA
	PGK1: sh-CUX1 #2 vs. sh-Scb; IMR32	5	0.0227	
	PGK1: E64D vs. DMSO; IMR32	5	0.0117	Student's unpaired *t*-test
	ENO1: CUX1 vs. Mock ; SH-SY5Y	5	0.0115	Student's unpaired *t*-test
	ENO1: sh-CUX1 #1 vs. sh-Scb; SH-SY5Y	5	0.0126	One-way ANOVA
	ENO1: sh-CUX1 #2 vs. sh-Scb; SH-SY5Y	5	0.0114	
	ENO1: E64D vs. DMSO; SH-SY5Y	5	0.0201	Student's unpaired *t*-test
	GPI: CUX1 vs. Mock ; SH-SY5Y	5	0.0105	Student's unpaired *t*-test
	GPI: sh-CUX1 #1 vs. sh-Scb; SH-SY5Y	5	0.0324	One-way ANOVA
	GPI: sh-CUX1 #2 vs. sh-Scb; SH-SY5Y	5	0.0334	
	GPI: E64D vs. DMSO; SH-SY5Y	5	0.0107	Student's unpaired *t*-test
	PGK1: CUX1 vs. Mock ; SH-SY5Y	5	0.0108	Student's unpaired *t*-test
	PGK1: sh-CUX1 #1 vs. sh-Scb; SH-SY5Y	5	0.0026	One-way ANOVA
	PGK1: sh-CUX1 #2 vs. sh-Scb; SH-SY5Y	5	0.0184	
	PGK1: E64D vs. DMSO; SH-SY5Y	5	0.0109	Student's unpaired *t*-test
Figure 1G	ECAR: CUX1 vs. Mock	5	0.0124	Student's unpaired *t*-test
	ECAR: sh-CUX1 #1 vs. sh-Scb	5	0.0382	One-way ANOVA
	ECAR: sh-CUX1 #2 vs. sh-Scb	5	0.0478	
	ECAR: E64D vs. DMSO	5	0.0247	Student's unpaired *t*-test
	OCR: CUX1 vs. Mock	5	0.1091	Student's unpaired *t*-test
	OCR: sh-CUX1 #1 vs. sh-Scb	5	0.0158	One-way ANOVA
	OCR: sh-CUX1 #2 vs. sh-Scb	5	0.0159	
	OCR: E64D vs. DMSO	5	0.0135	Student's unpaired *t*-test
Figure 2C	MCF 10A vs. HEK293	6	0.5943	One-way ANOVA
	HeLa vs. HEK293	6	0.0103	
	SH-SY5Y vs. HEK293	6	0.0108	
	IMR32 vs. HEK293	6	0.0124	
	SK-N-AS vs. HEK293	6	0.0331	
	LoVo vs. HEK293	6	0.0115	
	PC-3 vs. HEK293	6	0.0124	

Figure 2G	Neuroblastoma: NB vs. FAM; circ-CUX1	54 vs.13	0.0011	Student's unpaired *t*-test
	Colon cancer: Cancer vs. Normal; circ-CUX1	20 vs. 20	0.0082	
	Prostate cancer: Cancer vs. Normal; circ-CUX1	20 vs. 20	0.0065	
	Neuroblastoma: CUX1 vs. circ-CUX1	54	< 0.0001	Pearson's correlation coefficient
	Colon cancer: CUX1 vs. circ-CUX1	20	< 0.0001	
	Prostate cancer: CUX1 vs. circ-CUX1	20	0.0023	
Figure 3A	circ-CUX1 vs. Mock ; IMR32	5	0.0101	Student's unpaired *t*-test
	sh-circ-CUX1 #1 vs. sh-Scb; IMR32	5	0.0105	One-way ANOVA
	sh-circ-CUX1 #2 vs. sh-Scb; IMR32	5	0.0195	
	circ-CUX1 vs. Mock ; SH-SY5Y	5	0.0209	Student's unpaired *t*-test
	sh-circ-CUX1 #1 vs. sh-Scb; SH-SY5Y	5	0.0128	One-way ANOVA
	sh-circ-CUX1 #2 vs. sh-Scb; SH-SY5Y	5	0.0136	
Figure 3B	circ-CUX1 vs. Mock ; IMR32	5	0.0101	Student's unpaired *t*-test
	sh-circ-CUX1 #1 vs. sh-Scb; IMR32	5	0.0152	One-way ANOVA
	sh-circ-CUX1 #2 vs. sh-Scb; IMR32	5	0.0164	
	circ-CUX1 vs. Mock ; SH-SY5Y	5	0.0102	Student's unpaired *t*-test
	sh-circ-CUX1 #1 vs. sh-Scb; SH-SY5Y	5	0.0171	One-way ANOVA
	sh-circ-CUX1 #2 vs. sh-Scb; SH-SY5Y	5	0.0212	
Figure 3C	Tumor volume: circ-CUX1 vs. Mock; week1	5	0.1485	Student's unpaired *t*-test
	Tumor volume: circ-CUX1 vs. Mock; week2	5	0.0352	
	Tumor volume: circ-CUX1 vs. Mock; week3	5	0.0275	
	Tumor volume: circ-CUX1 vs. Mock; week4	5	0.0147	
	Tumor volume: sh-circ-CUX1 #1 vs. sh-Scb; week1	5	0.2134	
	Tumor volume: sh-circ-CUX1 #1 vs. sh-Scb; week2	5	0.03892	
	Tumor volume: sh-circ-CUX1 #1 vs. sh-Scb; week3	5	0.0284	
	Tumor volume: sh-circ-CUX1 #1 vs. sh-Scb; week4	5	0.0156	
	Tumor volume: circ-CUX1 vs. Mock	5	0.0116	One-way ANOVA
	Tumor volume: sh-circ-CUX1 #1 vs. sh-Scb	5	0.0127	
	Tumor weight: circ-CUX1 vs. Mock	5	0.0103	Student's unpaired *t*-test
	Tumor weight: sh-circ-CUX1 #1 vs. sh-Scb	5	0.0104	
Figure 3D	Ki-67: circ-CUX1 vs. Mock	5	0.0092	Student's unpaired *t*-test
	Ki-67: sh-circ-CUX1 #1 vs. sh-Scb	5	0.0041	
	CD31: circ-CUX1 vs. Mock	5	0.0102	
	CD31: sh-circ-CUX1 #1 vs. sh-Scb	5	0.0091	
Figure 3E	Glucose: circ-CUX1 vs. Mock	5	0.0013	Student's unpaired *t*-test
	Glucose: sh-circ-CUX1 #1 vs. sh-Scb	5	0.0055	
	Lactate: circ-CUX1 vs. Mock	5	0.0052	
	Lactate: sh-circ-CUX1 #1 vs. sh-Scb	5	0.0044	
	ATP: circ-CUX1 vs. Mock	5	0.0061	
	ATP: sh-circ-CUX1 #1 vs. sh-Scb	5	0.0046	
Figure 3F	Metastasis: circ-CUX1 vs. Mock	5	0.0033	Student's unpaired *t*-test
	Metastasis: sh-circ-CUX1 #1 vs. sh-Scb	5	0.0021	
	Survival: circ-CUX1 vs. Mock	5	< 0.0001	Log-rank test
	Survival: sh-circ-CUX1 #1 vs. sh-Scb	5	< 0.0001	
Figure 4C	circ-CUX1: EWSR1 vs. IgG; Untreated	5	0.0207	Student's unpaired *t*-test
	circ-CUX1: EWSR1 vs. IgG; Mock	5	0.0261	
	circ-CUX1: EWSR1 vs. IgG; circ-CUX1	5	0.0105	
	circ-CUX1: circ-CUX1 vs. Mock; EWSR1	5	0.0015	
	P200: EWSR1 vs. IgG; Untreated	5	0.152	
	P200: EWSR1 vs. IgG; Mock	5	0.8123	
	P200: EWSR1 vs. IgG; circ-CUX1	5	0.4773	
	CASP: EWSR1 vs. IgG; Untreated	5	0.9672	
	CASP: EWSR1 vs. IgG; Mock	5	0.716	
	CASP: EWSR1 vs. IgG; circ-CUX1	5	0.9311	
Figure 5E	CUX1: circ-CUX1+sh-Scb vs. Mock+sh-Scb	5	0.0113	One-way ANOVA
	CUX1: Mock+sh-EWSR1 #1 vs. Mock+sh-Scb	5	0.0101	
	CUX1: circ-CUX1+sh-EWSR1 #1. Mock+sh-Scb	5	0.4971	
	CUX1: Mock+sh-EWSR1 #2 vs. Mock+sh-Scb	5	0.0123	
	CUX1: circ-CUX1+sh-EWSR1 #2 vs. Mock+sh-Scb	5	0.8468	
	S100A9: circ-CUX1+sh-Scb vs. Mock+sh-Scb	5	0.0113	
	S100A9: Mock+sh-EWSR1 #1 vs. Mock+sh-Scb	5	0.0173	
	S100A9: circ-CUX1+sh-EWSR1 #1. Mock+sh-Scb	5	0.7538	
	S100A9: Mock+sh-EWSR1 #2 vs. Mock+sh-Scb	5	0.0237	
	S100A9: circ-CUX1+sh-EWSR1 #2 vs. Mock+sh-Scb	5	0.4912	
	MUC4: circ-CUX1+sh-Scb vs. Mock+sh-Scb	5	0.0119	
	MUC4: Mock+sh-EWSR1 #1 vs. Mock+sh-Scb	5	0.0215	
	MUC4: circ-CUX1+sh-EWSR1 #1. Mock+sh-Scb	5	0.6061	
	MUC4: Mock+sh-EWSR1 #2 vs. Mock+sh-Scb	5	0.0247	
	MUC4: circ-CUX1+sh-EWSR1 #2 vs. Mock+sh-Scb	5	0.5858	
	KLF10: circ-CUX1+sh-Scb vs. Mock+sh-Scb	5	0.0154	
	KLF10: Mock+sh-EWSR1 #1 vs. Mock+sh-Scb	5	0.0112	
	KLF10: circ-CUX1+sh-EWSR1 #1. Mock+sh-Scb	5	0.3012	
	KLF10: Mock+sh-EWSR1 #2 vs. Mock+sh-Scb	5	0.0112	
	KLF10: circ-CUX1+sh-EWSR1 #2 vs. Mock+sh-Scb	5	0.3208	
	TXNIP: circ-CUX1+sh-Scb vs. Mock+sh-Scb	5	0.0209	
	TXNIP: Mock+sh-EWSR1 #1 vs. Mock+sh-Scb	5	0.0175	
	TXNIP: circ-CUX1+sh-EWSR1 #1. Mock+sh-Scb	5	0.7177	
	TXNIP:Mock+sh-EWSR1 #2 vs. Mock+sh-Scb	5	0.0205	
	TXNIP: circ-CUX1+sh-EWSR1 #2 vs. Mock+sh-Scb	5	0.7366	
Figure 5F	Wild type: circ-CUX1+sh-Scb vs. Mock+sh-Scb	5	0.0103	One-way ANOVA
	Wild type: Mock+sh-EWSR1 #1 vs. Mock+sh-Scb	5	0.0108	
	Wild type: circ-CUX1+sh-EWSR1 #1. Mock+sh-Scb	5	0.3841	
	Wild type: Mock+sh-MAZ #2 vs. Mock+sh-Scb	5	0.0211	
	Wild type: circ-CUX1+sh-MAZ #2 vs. Mock+sh-Scb	5	0.606	
	Mutation: circ-CUX1+sh-Scb vs. Mock+sh-Scb	5	0.3156	
	Mutation: Mock+sh-EWSR1 #1 vs. Mock+sh-Scb	5	0.9182	
	Mutation: circ-CUX1+sh-EWSR1 #1. Mock+sh-Scb	5	0.6803	
	Mutation: Mock+sh-MAZ #2 vs. Mock+sh-Scb	5	0.4821	
	Mutation: circ-CUX1+sh-MAZ #2 vs. Mock+sh-Scb	5	0.6796	
Figure 5G	EWSR1: circ-CUX1+sh-Scb vs. Mock+sh-Scb	5	0.8149	One-way ANOVA
	EWSR1: Mock+sh-EWSR1 #1 vs. Mock+sh-Scb	5	0.0172	
	EWSR1: circ-CUX1+sh-EWSR1 #1. Mock+sh-Scb	5	0.0179	
	EWSR1: Mock+sh-MAZ #2 vs. Mock+sh-Scb	5	0.6123	
	EWSR1: circ-CUX1+sh-MAZ #2 vs. Mock+sh-Scb	5	0.6091	
	MAZ: circ-CUX1+sh-Scb vs. Mock+sh-Scb	5	0.9561	

	MAZ: Mock+sh-EWSR1 #1 vs. Mock+sh-Scb	5	0.9231	
	MAZ: circ-CUX1+sh-EWSR1 #1. Mock+sh-Scb	5	0.8259	
	MAZ: Mock+sh-MAZ #2 vs. Mock+sh-Scb	5	0.0119	
	MAZ: circ-CUX1+sh-MAZ #2 vs. Mock+sh-Scb	5	0.0191	
	CUX1: circ-CUX1+sh-Scb vs. Mock+sh-Scb	5	0.0103	
	CUX1: Mock+sh-EWSR1 #1 vs. Mock+sh-Scb	5	0.0114	
	CUX1: circ-CUX1+sh-EWSR1 #1. Mock+sh-Scb	5	0.4245	
	CUX1: Mock+sh-MAZ #2 vs. Mock+sh-Scb	5	0.0201	
	CUX1: circ-CUX1+sh-MAZ #2 vs. Mock+sh-Scb	5	0.1192	
	S100A9: circ-CUX1+sh-Scb vs. Mock+sh-Scb	5	0.0103	
	S100A9: Mock+sh-EWSR1 #1 vs. Mock+sh-Scb	5	0.0382	
	S100A9: circ-CUX1+sh-EWSR1 #1. Mock+sh-Scb	5	0.2996	
	S100A9: Mock+sh-MAZ #2 vs. Mock+sh-Scb	5	0.0193	
	S100A9: circ-CUX1+sh-MAZ #2 vs. Mock+sh-Scb	5	0.5871	
	MUC4: circ-CUX1+sh-Scb vs. Mock+sh-Scb	5	0.0201	One-way ANOVA
	MUC4: Mock+sh-EWSR1 #1 vs. Mock+sh-Scb	5	0.0144	
	MUC4: circ-CUX1+sh-EWSR1 #1. Mock+sh-Scb	5	0.5051	
	MUC4: Mock+sh-MAZ #2 vs. Mock+sh-Scb	5	0.0104	
	MUC4: circ-CUX1+sh-MAZ #2 vs. Mock+sh-Scb	5	0.3411	
	KLF10: circ-CUX1+sh-Scb vs. Mock+sh-Scb	5	0.0175	
	KLF10: Mock+sh-EWSR1 #1 vs. Mock+sh-Scb	5	0.0102	
	KLF10: circ-CUX1+sh-EWSR1 #1. Mock+sh-Scb	5	0.5728	
	KLF10: Mock+sh-MAZ #2 vs. Mock+sh-Scb	5	0.0101	
	KLF10: circ-CUX1+sh-MAZ #2 vs. Mock+sh-Scb	5	0.2974	
	TXNIP: circ-CUX1+sh-Scb vs. Mock+sh-Scb	5	0.0101	
	TXNIP: Mock+sh-EWSR1 #1 vs. Mock+sh-Scb	5	0.0112	
	TXNIP: circ-CUX1+sh-EWSR1 #1. Mock+sh-Scb	5	0.5935	
	TXNIP:Mock+sh-MAZ #2 vs. Mock+sh-Scb	5	0.0104	
	TXNIP: circ-CUX1+sh-MAZ #2 vs. Mock+sh-Scb	5	0.5156	
Figure 6D	circ-CUX1: EIP-22 vs. CTLP	5	0.0102	
	Pri-miR-222: EIP-22 vs. CTLP	5	0.8325	Student's unpaired *t*-test
	circACC1: EIP-22 vs. CTLP	5	0.9765	
Figure 6E	EIP-22 vs. CTLP	5	0.0018	Student's unpaired *t*-test
Figure 6F	EIP-22 vs. CTLP	5	0.0016	Student's unpaired *t*-test
Figure 6G	Tumor volume: EIP-22 vs.CTLP ; Week 1	5	0.783	
	Tumor volume: EIP-22 vs.CTLP ; Week 2	5	0.0382	
	Tumor volume: EIP-22 vs.CTLP ; Week 3	5	0.0184	Student's unpaired *t*-test
	Tumor volume: EIP-22 vs.CTLP ; Week 4	5	0.0163	
	Tumor weight: EIP-22 vs.CTLP	5	0.0012	
Figure 6H	Metastasis: EIP-22 vs. CTLP	5	< 0.0001	Student's unpaired *t*-test
	Survival: EIP-22 vs. CTLP	5	0.0018	Log-rank test
Figure EV1C	has_circ_0132813: NB vs. FAM	20 vs. 13	0.0109	
	has_circ_0132814: NB vs. FAM	20 vs. 13	0.2859	
	has_circ_0132817: NB vs. FAM	20 vs. 13	0.0157	
	has_circ_0005253: NB vs. FAM	20 vs. 13	0.0123	Student's unpaired *t*-test
	has_circ_0006501: NB vs. FAM	20 vs. 13	0.4649	
	has_circ_0081618: NB vs. FAM	20 vs. 13	0.5582	
	has_circ_0081622: NB vs. FAM	20 vs. 13	0.2846	
Figure EV1D	IMR32: p110 CUX1 vs. Mock	5	0.0103	Student's unpaired *t*-test
	IMR32: sh-CUX1 vs. sh-Scb	5	0.0153	
	IMR32: sh-has_circ_0132813 vs. sh-Scb	5	0.0107	One-way ANOVA
	IMR32: sh-has_circ_0132817 vs. sh-Scb	5	0.2449	
	IMR32: sh-has_circ_0005253 vs. sh-Scb	5	0.2535	
	SH-SY5Y: p110 CUX1 vs. Mock	5	0.0143	Student's unpaired *t*-test
	SH-SY5Y: sh-CUX1 vs. sh-Scb	5	0.0109	
	SH-SY5Y: sh-has_circ_0132813 vs. sh-Scb	5	0.0243	One-way ANOVA
	SH-SY5Y: sh-has_circ_0132817 vs. sh-Scb	5	0.3492	
	SH-SY5Y: sh-has_circ_0005253 vs. sh-Scb	5	0.3031	
Figure EV1E	Sroma: Poor vs. Rich	54	0.0083	
	INSS stage: 3+4 vs. 1+2+4s	54	0.0017	Student's unpaired *t*-test
	MYCN amplification: Yes vs. No	54	0.1532	
Figure EV1F	Survival: High vs. Low	54	0.0062	Log-rank test
Figure EV2A	circ-CUX1 vs. Mock; IMR32	5	0.0201	
	circ-CUX1-Mut vs. Mock; IMR32	5	0.5061	
	sh-circ-CUX1 #1 vs. sh-Scb; IMR32	5	0.0108	
	sh-circ-CUX1 #2 vs. sh-Scb; IMR32	5	0.0101	
	circ-CUX1 vs. Mock; SH-SY5Y	5	0.0102	
	circ-CUX1-Mut vs. Mock; SH-SY5Y	5	0.8314	
	sh-circ-CUX1 #1 vs. sh-Scb; SH-SY5Y	5	0.0106	
	sh-circ-CUX1 #2 vs. sh-Scb; SH-SY5Y	5	0.0225	
	circ-CUX1 vs. Mock; LoVo	5	0.0207	One-way ANOVA
	circ-CUX1-Mut vs. Mock; LoVo	5	0.6098	
	sh-circ-CUX1 #1 vs. sh-Scb; LoVo	5	0.0114	
	sh-circ-CUX1 #2 vs. sh-Scb; LoVo	5	0.0188	
	circ-CUX1 vs. Mock; PC-3	5	0.0101	
	circ-CUX1-Mut vs. Mock; PC-3	5	0.5661	
	sh-circ-CUX1 #1 vs. sh-Scb; PC-3	5	0.0122	
	sh-circ-CUX1 #2 vs. sh-Scb; PC-3	5	0.0142	
Figure EV2B	circ-CUX1 vs. Mock; IMR32	5	0.0116	
	circ-CUX1-Mut vs. Mock; IMR32	5	0.3438	
	sh-circ-CUX1 #1 vs. sh-Scb; IMR32	5	0.0316	
	sh-circ-CUX1 #2 vs. sh-Scb; IMR32	5	0.0412	
	circ-CUX1 vs. Mock; SH-SY5Y	5	0.0147	
	circ-CUX1-Mut vs. Mock; SH-SY5Y	5	0.5569	
	sh-circ-CUX1 #1 vs. sh-Scb; SH-SY5Y	5	0.0456	
	sh-circ-CUX1 #2 vs. sh-Scb; SH-SY5Y	5	0.0469	
	circ-CUX1 vs. Mock; LoVo	5	0.0103	One-way ANOVA
	circ-CUX1-Mut vs. Mock; LoVo	5	0.8911	
	sh-circ-CUX1 #1 vs. sh-Scb; LoVo	5	0.0259	
	sh-circ-CUX1 #2 vs. sh-Scb; LoVo	5	0.0298	
	circ-CUX1 vs. Mock; PC-3	5	0.0101	
	circ-CUX1-Mut vs. Mock; PC-3	5	0.8548	
	sh-circ-CUX1 #1 vs. sh-Scb; PC-3	5	0.0281	
	sh-circ-CUX1 #2 vs. sh-Scb; PC-3	5	0.0349	
Figure EV2C	p200: circ-CUX1 vs. Mock; IMR32	5	0.0169	One-way ANOVA

	p200: circ-CUX1-Mut vs. Mock; IMR32	5	0.6223	One-way ANOVA
	p200: sh-circ-CUX1 #1 vs. sh-Scb; IMR32	5	0.0191	
	p200: sh-circ-CUX1 #2 vs. sh-Scb; IMR32	5	0.0251	
	CASP: circ-CUX1 vs. Mock; IMR32	5	0.0601	
	CASP: circ-CUX1-Mut vs. Mock; IMR32	5	0.4537	
	CASP: sh-circ-CUX1 #1 vs. sh-Scb; IMR32	5	0.1993	
	CASP: sh-circ-CUX1 #2 vs. sh-Scb; IMR32	5	0.1809	
	p200: circ-CUX1 vs. Mock; SH-SY5Y	5	0.0114	
	p200: circ-CUX1-Mut vs. Mock; SH-SY5Y	5	0.8623	
	p200: sh-circ-CUX1 #1 vs. sh-Scb; SH-SY5Y	5	0.0136	
	p200: sh-circ-CUX1 #2 vs. sh-Scb; SH-SY5Y	5	0.0175	
	CASP: circ-CUX1 vs. Mock; SH-SY5Y	5	0.2211	
	CASP: circ-CUX1-Mut vs. Mock; SH-SY5Y	5	0.5939	
	CASP: sh-circ-CUX1 #1 vs. sh-Scb; SH-SY5Y	5	0.3807	
	CASP: sh-circ-CUX1 #2 vs. sh-Scb; SH-SY5Y	5	0.3655	
Figure EV2D	circ-CUX1 vs. Mock; IMR32	5	0.7686	One-way ANOVA
	circ-CUX1 vs. Mock; SH-SY5Y	5	0.8718	
Figure EV2E	ECAR: circ-CUX1 vs. Mock; IMR32	6	0.0103	One-way ANOVA
	ECAR: circ-CUX1-Mut vs. Mock; IMR32	6	0.4963	
	ECAR: sh-circ-CUX1 #1 vs. sh-Scb; IMR32	6	0.0119	
	ECAR: sh-circ-CUX1 #2 vs. sh-Scb; IMR32	6	0.0207	
	ECAR: circ-CUX1 vs. Mock; SH-SY5Y	6	0.0123	
	ECAR: circ-CUX1-Mut vs. Mock; SH-SY5Y	6	0.1772	
	ECAR: sh-circ-CUX1 #1 vs. sh-Scb; SH-SY5Y	6	0.0132	
	ECAR: sh-circ-CUX1 #2 vs. sh-Scb; SH-SY5Y	6	0.0238	
	ECAR: circ-CUX1 vs. Mock; LoVo	6	0.0103	
	ECAR: circ-CUX1-Mut vs. Mock; LoVo	6	0.8904	
	ECAR: sh-circ-CUX1 #1 vs. sh-Scb; LoVo	6	0.0105	
	ECAR: sh-circ-CUX1 #2 vs. sh-Scb; LoVo	6	0.0106	
	ECAR: circ-CUX1 vs. Mock; PC-3	6	0.0216	
	ECAR: circ-CUX1-Mut vs. Mock; PC-3	6	0.4083	
	ECAR: sh-circ-CUX1 #1 vs. sh-Scb; PC-3	6	0.0144	
	ECAR: sh-circ-CUX1 #2 vs. sh-Scb; PC-3	6	0.0135	
	OCR: circ-CUX1 vs. Mock; IMR32	6	0.0118	
	OCR: circ-CUX1-Mut vs. Mock; IMR32	6	0.6143	
	OCR: sh-circ-CUX1 #1 vs. sh-Scb; IMR32	6	0.0201	
	OCR: sh-circ-CUX1 #2 vs. sh-Scb; IMR32	6	0.0102	
	OCR: circ-CUX1 vs. Mock; SH-SY5Y	6	0.0202	
	OCR: circ-CUX1-Mut vs. Mock; SH-SY5Y	6	0.9262	
	OCR: sh-circ-CUX1 #1 vs. sh-Scb; SH-SY5Y	6	0.0101	
	OCR: sh-circ-CUX1 #2 vs. sh-Scb; SH-SY5Y	6	0.0202	
	OCR: circ-CUX1 vs. Mock; LoVo	6	0.0121	
	OCR: circ-CUX1-Mut vs. Mock; LoVo	6	0.1872	
	OCR: sh-circ-CUX1 #1 vs. sh-Scb; LoVo	6	0.0101	
	OCR: sh-circ-CUX1 #2 vs. sh-Scb; LoVo	6	0.0103	
	OCR: circ-CUX1 vs. Mock; PC-3	6	0.0102	
	OCR: circ-CUX1-Mut vs. Mock; PC-3	6	0.4077	
	OCR: sh-circ-CUX1 #1 vs. sh-Scb; PC-3	6	0.0203	
	OCR: sh-circ-CUX1 #2 vs. sh-Scb; PC-3	6	0.0119	
Figure S1A	p200: NB vs.FAM	54 vs. 13	0.0125	One-way ANOVA
	p75: NB vs.FAM	55 vs. 13	0.8251	
	CASP: NB vs.FAM	56 vs. 13	0.5123	
	p200: SH-SY5Y vs.FAM	5	0.0165	
	p75: SH-SY5Y vs.FAM	5	0.7658	
	CASP: SH-SY5Y vs.FAM	5	0.4698	
	p200: IMR32 vs.FAM	5	0.0201	
	p75: IMR32 vs.FAM	5	0.8123	
	CASP: IMR32 vs.FAM	5	0.4536	
	p200: SK-N-AS vs.FAM	5	0.0123	
	p75: SK-N-AS vs.FAM	5	0.7562	
	CASP: SK-N-AS vs.FAM	5	0.4635	
Figure S1B	Sroma: Poor vs. Rich	54	0.0002	Student's unpaired *t*-test
	INSS stage: 3+4 vs. 1+2+4s	54	0.007	
	MYCN amplification: Yes vs. No	54	0.56	
Figure S1C	CUX1: CUX1 vs. Mock	5	0.0125	Student's unpaired *t*-test
	CUX1: sh-CUX1 #1 vs. sh-Scb	5	0.0109	One-way ANOVA
	CUX1: sh-CUX1 #2 vs. sh-Scb	5	0.0145	
	ALDOA: CUX1 vs. Mock	5	0.8808	Student's unpaired *t*-test
	ALDOA: sh-CUX1 #1 vs. sh-Scb	5	0.5218	One-way ANOVA
	ALDOA: sh-CUX1 #2 vs. sh-Scb	5	0.6773	
	ENO1: CUX1 vs. Mock	5	0.0242	Student's unpaired *t*-test
	ENO1: sh-CUX1 #1 vs. sh-Scb	5	0.0131	One-way ANOVA
	ENO1: sh-CUX1 #2 vs. sh-Scb	5	0.0133	
	GAPDH: CUX1 vs. Mock	5	0.7163	Student's unpaired *t*-test
	GAPDH: sh-CUX1 #1 vs. sh-Scb	5	0.3169	One-way ANOVA
	GAPDH: sh-CUX1 #2 vs. sh-Scb	5	0.9711	
	GPI: CUX1 vs. Mock	5	0.0121	Student's unpaired *t*-test
	GPI: sh-CUX1 #1 vs. sh-Scb	5	0.0152	One-way ANOVA
	GPI: sh-CUX1 #2 vs. sh-Scb	5	0.0179	
	HK2: CUX1 vs. Mock	5	0.9301	Student's unpaired *t*-test
	HK2: sh-CUX1 #1 vs. sh-Scb	5	0.6965	One-way ANOVA
	HK2: sh-CUX1 #2 vs. sh-Scb	5	0.6821	
	LDHA: CUX1 vs. Mock	5	0.896	Student's unpaired *t*-test
	LDHA: sh-CUX1 #1 vs. sh-Scb	5	0.7627	One-way ANOVA
	LDHA: sh-CUX1 #2 vs. sh-Scb	5	0.9609	
	PGK1: CUX1 vs. Mock	5	0.0105	Student's unpaired *t*-test
	PGK1: sh-CUX1 #1 vs. sh-Scb	5	0.0216	One-way ANOVA
	PGK1: sh-CUX1 #2 vs. sh-Scb	5	0.0121	
	PKM: CUX1 vs. Mock	5	0.1699	Student's unpaired *t*-test
	PKM: sh-CUX1 #1 vs. sh-Scb	5	0.3041	One-way ANOVA
	PKM: sh-CUX1 #2 vs. sh-Scb	5	0.3004	
Figure S1F	CASP: sh-CASP #1 vs. sh-Scb	5	0.0225	One-way ANOVA
	CASP: sh-CASP #2 vs. sh-Scb	5	0.0285	
	CASP: sh-CASP #1 vs. sh-Scb	5	0.9794	
	CASP: sh-CASP #2 vs. sh-Scb	5	0.9658	

	CASP: sh-CASP #1 vs. sh-Scb	5	0.6255	
	CASP: sh-CASP #2 vs. sh-Scb	5	0.7397	One-way ANOVA
	CASP: sh-CASP #1 vs. sh-Scb	5	0.6702	
	CASP: sh-CASP #2 vs. sh-Scb	5	0.6081	
Figure S2A	Glucose: p110 CUX1+PBS vs. Mock+PBS	5	0.0142	
	Glucose: Mock+2-DG vs. Mock+PBS	5	0.0104	
	Glucose: p110 CUX1+2-DG vs. Mock+PBS	5	0.1953	One-way ANOVA
	Glucose: sh-CUX1 #1 vs. sh-Scb	5	0.0104	
	Glucose: sh-CUX1 #2 vs. sh-Scb	5	0.0158	
	Glucose: E64D vs. DMSO	5	0.0264	Student's unpaired *t*-test
	Lactate: p110 CUX1+PBS vs. Mock+PBS	5	0.0133	
	Lactate: Mock+2-DG vs. Mock+PBS	5	0.0104	
	Lactate: p110 CUX1+2-DG vs. Mock+PBS	5	0.3761	One-way ANOVA
	Lactate: sh-CUX1 #1 vs. sh-Scb	5	0.0131	
	Lactate: sh-CUX1 #2 vs. sh-Scb	5	0.0172	
	Lactate: E64D vs. DMSO	5	0.0176	Student's unpaired *t*-test
	ATP: p110 CUX1+PBS vs. Mock+PBS	5	0.0119	
	ATP: Mock+2-DG vs. Mock+PBS	5	0.0146	
	ATP: p110 CUX1+2-DG vs. Mock+PBS	5	0.5551	One-way ANOVA
	ATP: sh-CUX1 #1 vs. sh-Scb	5	0.0321	
	ATP: sh-CUX1 #2 vs. sh-Scb	5	0.0473	
	ATP: E64D vs. DMSO	5	0.0322	Student's unpaired *t*-test
Figure S2D	Colonies: p110 CUX1+DMSO vs. Mock+DMSO	5	0.0102	
	Colonies: Mock+2-DG vs. Mock+DMSO	5	0.0106	One-way ANOVA
	Colonies: p110 CUX1+2-DG vs. Mock+DMSO	5	0.2795	
	Colonies: sh-CUX1 #1 vs. sh-Scb	5	0.0105	Student's unpaired *t*-test
	Colonies: E64D vs. DMSO	5	0.0204	
	Invasion: p110 CUX1+DMSO vs. Mock+DMSO	5	0.0101	
	Invasion: Mock+2-DG vs. Mock+DMSO	5	0.0171	One-way ANOVA
	Invasion: p110 CUX1+2-DG vs. Mock+DMSO	5	0.4798	
	Invasion: sh-CUX1 #1 vs. sh-Scb	5	0.0103	Student's unpaired *t*-test
	Invasion: E64D vs. DMSO	5	0.0129	
Figure S2E	Neuroblastoma: ENO1 vs. CUX1	88	0.0098	
	Neuroblastoma: GPI vs. CUX1	88	0.0056	
	Neuroblastoma: PGK1 vs. CUX1	88	0.0022	
	Colon cancer: ENO1 vs. CUX1	80	2.6×10^{-4}	
	Colon cancer: GPI vs. CUX1	80	5.7×10^{-5}	Pearson’s correlation coefficient
	Colon cancer: PGK1 vs. CUX1	80	1.9×10^{-5}	
	Prostate cancer: ENO1 vs. CUX1	89	1.2×10^{-6}	
	Prostate cancer: GPI vs. CUX1	89	9.1×10^{-11}	
	Prostate cancer: PGK1 vs. CUX1	89	3.8×10^{-8}	
Figure S3	Neuroblastoma Survival: CUX1 high vs. CUX1 low	88	0.028	
	Neuroblastoma Survival: ENO1 high vs. ENO1 low	88	0.0032	
	Neuroblastoma Survival: GPI high vs. GPI low	88	5.3×10^{-8}	
	Neuroblastoma Survival: PGK1 high vs. PGK1 low	88	1.5×10^{-7}	
	Neuroblastoma Survival: CUX1 high vs. CUX1 low	498	0.0066	
	Neuroblastoma Survival: ENO1 high vs. ENO1 low	498	3.8×10^{-14}	
	Neuroblastoma Survival: GPI high vs. GPI low	498	0.0014	
	Neuroblastoma Survival: PGK1 high vs. PGK1 low	498	3.0×10^{-23}	
	Colon cancer Survival: CUX1 high vs. CUX1 low	243	0.022	
	Colon cancer Survival: ENO1 high vs. ENO1 low	243	0.014	
	Colon cancer Survival: GPI high vs. GPI low	243	7.9×10^{-4}	
	Colon cancer Survival: PGK1 high vs. PGK1 low	243	8.9×10^{-3}	
	Prostate cancer Survival: CUX1 high vs. CUX1 low	140	1.1×10^{-2}	Log-rank test
	Prostate cancer Survival: ENO1 high vs. ENO1 low	140	1.5×10^{-3}	
	Prostate cancer Survival: GPI high vs. GPI low	140	2.0×10^{-4}	
	Prostate cancer Survival: PGK1 high vs. PGK1 low	140	1.1×10^{-2}	
	Gastric cancer Survival: CUX1 high vs. CUX1 low	876	0.0019	
	Gastric cancer Survival: ENO1 high vs. ENO1 low	876	0.0012	
	Gastric cancer Survival: GPI high vs. GPI low	876	1.7×10^{-9}	
	Gastric cancer Survival: PGK1 high vs. PGK1 low	876	1.2×10^{-4}	
	Breast cancer Survival: CUX1 high vs. CUX1 low	1402	1.0×10^{-5}	
	Breast cancer Survival: ENO1 high vs. ENO1 low	1402	7.4×10^{-6}	
	Breast cancer Survival: GPI high vs. GPI low	1402	0.0024	
	Breast cancer Survival: PGK1 high vs. PGK1 low	1402	4.9×10^{-13}	
Figure S4A	NB vs. Normal	180	0.5919	
	Death: Yes vs. No	177	0.3114	Student's unpaired *t*-test
	MYCN amplification: Yes vs. No	177	0.8916	
	INSS stage: 3+4 vs. 1+2+4s	188	0.1977	
Figure S4B	Survival: High vs. Low	114	0.3799	Log-rank test
Figure S5A	Linear: circ-CUX1 vs. Mock	6	0.0246	
	Linear: circ-CUX1-Mut vs. Mock	6	0.0101	
	Circular: circ-CUX1 vs. Mock	6	0.0156	One-way ANOVA
	Circular: circ-CUX1-Mut vs. Mock	6	0.5785	
	Circular: circ-CUX1+RNase R vs. Mock+RNase R	6	0.0251	
	Circular: circ-CUX1-Mut+RNase R vs. Mock+RNase R	6	0.6131	
Figure S5B	circ-CUX1 RNAi reporter: sh-circ-CUX1 #1 vs. sh-Scb; IMR32	6	0.0106	
	circ-CUX1 RNAi reporter: sh-circ-CUX1 #2 vs. sh-Scb; IMR32	6	0.0205	
	circ-CUX1 RNAi reporter: sh-CUX1 #1 vs. sh-Scb; IMR32	6	0.7771	
	circ-CUX1 RNAi reporter: sh-CUX1 #2 vs. sh-Scb; IMR32	6	0.5701	
	circ-CUX1 RNAi reporter: sh-circ-CUX1 #1 vs. sh-Scb; SH-SY5Y	6	0.0109	
	circ-CUX1 RNAi reporter: sh-circ-CUX1 #2 vs. sh-Scb; SH-SY5Y	6	0.0127	
	circ-CUX1 RNAi reporter: sh-CUX1 #1 vs. sh-Scb; SH-SY5Y	6	0.7333	
	circ-CUX1 RNAi reporter: sh-CUX1 #2 vs. sh-Scb; SH-SY5Y	6	0.8157	
	circ-CUX1 RNAi reporter: sh-circ-CUX1 #1 vs. sh-Scb; LoVo	6	0.0106	
	circ-CUX1 RNAi reporter: sh-circ-CUX1 #2 vs. sh-Scb; LoVo	6	0.0214	One-way ANOVA
	circ-CUX1 RNAi reporter: sh-CUX1 #1 vs. sh-Scb; LoVo	6	0.5467	
	circ-CUX1 RNAi reporter: sh-CUX1 #2 vs. sh-Scb; LoVo	6	0.8397	
	circ-CUX1 RNAi reporter: sh-circ-CUX1 #1 vs. sh-Scb; PC-3	6	0.0201	
	circ-CUX1 RNAi reporter: sh-circ-CUX1 #2 vs. sh-Scb; PC-3	6	0.0121	
	circ-CUX1 RNAi reporter: sh-CUX1 #1 vs. sh-Scb; PC-3	6	0.2758	
	circ-CUX1 RNAi reporter: sh-CUX1 #2 vs. sh-Scb; PC-3	6	0.0766	
	p200 RNAi reporter: sh-circ-CUX1 #1 vs. sh-Scb; IMR32	6	0.5962	
	p200 RNAi reporter: sh-circ-CUX1 #2 vs. sh-Scb; IMR32	6	0.6415	
	p200 RNAi reporter: sh-CUX1 #1 vs. sh-Scb; IMR32	6	0.0121	

	p200 RNAi reporter: sh-CUX1 #2 vs. sh-Scb; IMR32	6	0.0152	
	p200 RNAi reporter: sh-circ-CUX1 #1 vs. sh-Scb; SH-SY5Y	6	0.8523	
	p200 RNAi reporter: sh-circ-CUX1 #2vs. sh-Scb; SH-SY5Y	6	0.5647	
	p200 RNAi reporter: sh-CUX1 #1 vs. sh-Scb; SH-SY5Y	6	0.0201	
	p200 RNAi reporter: sh-CUX1 #2 vs. sh-Scb; SH-SY5Y	6	0.0131	
	p200 RNAi reporter: sh-circ-CUX1 #1 vs. sh-Scb; LoVo	6	0.4391	
	p200 RNAi reporter: sh-circ-CUX1 #2 vs. sh-Scb; LoVo	6	0.6213	One-way ANOVA
	p200 RNAi reporter: sh-CUX1 #1 vs. sh-Scb; LoVo	6	0.0131	
	p200 RNAi reporter: sh-CUX1 #2 vs. sh-Scb; LoVo	6	0.0151	
	p200 RNAi reporter: sh-circ-CUX1 #1 vs. sh-Scb; PC-3	6	0.5127	
	p200 RNAi reporter: sh-circ-CUX1 #1 vs. sh-Scb; PC-3	6	0.8438	
	p200 RNAi reporter: sh-CUX1 #1 vs. sh-Scb; PC-3	6	0.0211	
	p200 RNAi reporter: sh-CUX1 #2 vs. sh-Scb; PC-3	6	0.0132	
Figure S5C	circ-CUX1: sh-circ-CUX1 #1 vs. sh-Scb; IMR32	5	0.0137	
	circ-CUX1: sh-circ-CUX1 #2 vs. sh-Scb; IMR32	5	0.0113	
	circ-CUX1: sh-CUX1 #1 vs. sh-Scb; IMR32	5	0.5641	
	circ-CUX1: sh-CUX1 #2 vs. sh-Scb; IMR32	5	0.9529	
	circ-CUX1: sh-circ-CUX1 #1 vs. sh-Scb; SH-SY5Y	5	0.0204	
	circ-CUX1: sh-circ-CUX1 #2 vs. sh-Scb; SH-SY5Y	5	0.0303	
	circ-CUX1: sh-CUX1 #1 vs. sh-Scb; SH-SY5Y	5	0.9556	
	circ-CUX1: sh-CUX1 #2 vs. sh-Scb; SH-SY5Y	5	0.6531	
	circ-CUX1: sh-circ-CUX1 #1 vs. sh-Scb; LoVo	5	0.0104	
	circ-CUX1: sh-circ-CUX1 #2 vs. sh-Scb; LoVo	5	0.0133	
	circ-CUX1: sh-CUX1 #1 vs. sh-Scb; LoVo	5	0.3431	
	circ-CUX1: sh-CUX1 #2 vs. sh-Scb; LoVo	5	0.4043	
	circ-CUX1: sh-circ-CUX1 #1 vs. sh-Scb; PC-3	5	0.0002	
	circ-CUX1: sh-circ-CUX1 #2 vs. sh-Scb; PC-3	5	0.0131	
	circ-CUX1: sh-CUX1 #1 vs. sh-Scb; PC-3	5	0.7124	
	circ-CUX1: sh-CUX1 #2 vs. sh-Scb; PC-3	5	0.3941	One-way ANOVA
	p200-CUX1: sh-circ-CUX1 #1 vs. sh-Scb; IMR32	5	0.7497	
	p200-CUX1: sh-circ-CUX1 #2 vs. sh-Scb; IMR32	5	0.4496	
	p200-CUX1: sh-CUX1 #1 vs. sh-Scb; IMR32	5	0.0131	
	p200-CUX1: sh-CUX1 #2 vs. sh-Scb; IMR32	5	0.0153	
	p200-CUX1: sh-circ-CUX1 #1 vs. sh-Scb; SH-SY5Y	5	0.5892	
	p200-CUX1: sh-circ-CUX1 #2 vs. sh-Scb; SH-SY5Y	5	0.9463	
	p200-CUX1: sh-CUX1 #1 vs. sh-Scb; SH-SY5Y	5	0.0201	
	p200-CUX1: sh-CUX1 #2 vs. sh-Scb; SH-SY5Y	5	0.0102	
	p200-CUX1: sh-circ-CUX1 #1 vs. sh-Scb; LoVo	5	0.6865	
	p200-CUX1: sh-circ-CUX1 #2 vs. sh-Scb; LoVo	5	0.7174	
	p200-CUX1: sh-CUX1 #1 vs. sh-Scb; LoVo	5	0.0303	
	p200-CUX1: sh-CUX1 #2 vs. sh-Scb; LoVo	5	0.0102	
	p200-CUX1: sh-circ-CUX1 #1 vs. sh-Scb; PC-3	5	0.7633	
	p200-CUX1: sh-circ-CUX1 #2 vs. sh-Scb; PC-3	5	0.8966	
	p200-CUX1: sh-CUX1 #1 vs. sh-Scb; PC-3	5	0.0104	
	p200-CUX1: sh-CUX1 #2 vs. sh-Scb; PC-3	5	0.0163	
Figure S6A	Glucose: circ-CUX1 vs. Mock+PBS	5	0.0125	
	Glucose: Mock+2-DG vs. Mock+PBS	5	0.0253	
	Glucose: circ-CUX1+2-DG vs. Mock+PBS	5	0.9615	
	Lactate: circ-CUX1 vs. Mock+PBS	5	0.0139	
	Lactate: Mock+2-DG vs. Mock+PBS	5	0.0257	One-way ANOVA
	Lactate: circ-CUX1+2-DG vs. Mock+PBS	5	0.7782	
	ATP: circ-CUX1 vs. Mock+PBS	5	0.0124	
	ATP: Mock+2-DG vs. Mock+PBS	5	0.0316	
	ATP: circ-CUX1+2-DG vs. Mock+PBS	5	0.8537	
Figure S7A	NB vs. FAM	54 vs. 13	< 0.0001	
	Stroma: Rich vs. Poor	54	0.0205	Student's unpaired *t*-test
	INSS stage: 3+4 vs. 1+2+4s	54	0.0097	
	MYCN amplification: Yes vs. No	54	0.6445	
Figure S7B	CUX1 vs. MAZ	54	< 0.0001	
	S100A9 vs. MAZ	54	< 0.0001	
	MUC4 vs. MAZ	54	< 0.0001	Pearson’s correlation coefficient
	KLF10 vs. MAZ	54	0.0002	
	TXNIP vs. MAZ	54	0.0004	
Figure S7C	EWSR1 high vs. EWSR1 low	88	0.0446	
	MAZ high vs. MAZ low	88	0.0066	
	S100A9 high vs. S100A9 low	88	0.025	Log-rank test
	MUC4 high vs. MUC4 low	88	0.001	
	KLF10 high vs. KLF10 low	88	0.031	
	TXNIP high vs. TXNIP low	88	0.041	
Figure S8A	CUX1: AS probe vs. S probe	5	0.0103	
	ENO1: AS probe vs. S probe	5	0.7676	
	GPI: AS probe vs. S probe	5	0.6213	
	PGK1: AS probe vs. S probe	5	0.8541	Student's unpaired *t*-test
	S100A9: AS probe vs. S probe	5	0.0205	
	MUC4: AS probe vs. S probe	5	0.0118	
	KLF10: AS probe vs. S probe	5	0.0188	
	TXNIP: AS probe vs. S probe	5	0.0214	
Figure S8B	CUX1: sh-Scb+EWSR1 vs. sh-Scb+Mock	5	0.0128	
	CUX1: sh-circ-CUX1 #1+Mock vs. sh-Scb+Mock	5	0.0163	
	CUX1: sh-circ-CUX1 #1+EWSR1 vs. sh-Scb+Mock	5	0.9945	
	CUX1: sh-circ-CUX1 #2+Mock vs. sh-Scb+Mock	5	0.0276	
	CUX1: sh-circ-CUX1 #2+EWSR1 vs. sh-Scb+Mock	5	0.8401	
	S100A9: sh-Scb+EWSR1 vs. sh-Scb+Mock	5	0.0151	
	S100A9: sh-circ-CUX1 #1+Mock vs. sh-Scb+Mock	5	0.0129	
	S100A9: sh-circ-CUX1 #1+EWSR1 vs. sh-Scb+Mock	5	0.6432	
	S100A9: sh-circ-CUX1 #2+Mock vs. sh-Scb+Mock	5	0.0398	One-way ANOVA
	S100A9: sh-circ-CUX1 #2+EWSR1 vs. sh-Scb+Mock	5	0.4773	
	MUC4: sh-Scb+EWSR1 vs. sh-Scb+Mock	5	0.0143	
	MUC4: sh-circ-CUX1 #1+Mock vs. sh-Scb+Mock	5	0.0242	
	MUC4: sh-circ-CUX1 #1+EWSR1 vs. sh-Scb+Mock	5	0.9048	
	MUC4: sh-circ-CUX1 #2+Mock vs. sh-Scb+Mock	5	0.0145	
	MUC4: sh-circ-CUX1 #2+EWSR1 vs. sh-Scb+Mock	5	0.8074	
	KLF10: sh-Scb+EWSR1 vs. sh-Scb+Mock	5	0.0119	
	KLF10: sh-circ-CUX1 #1+Mock vs. sh-Scb+Mock	5	0.0238	
	KLF10: sh-circ-CUX1 #1+EWSR1 vs. sh-Scb+Mock	5	0.6598	

	KLF10: sh-circ-CUX1 #2+Mock vs. sh-Scb+Mock	5	0.0147	One-way ANOVA
	KLF10: sh-circ-CUX1 #2+EWSR1 vs. sh-Scb+Mock	5	0.8646	
	TXNIP: sh-Scb+EWSR1 vs. sh-Scb+Mock	5	0.0145	
	TXNIP: sh-circ-CUX1 #1+Mock vs. sh-Scb+Mock	5	0.0167	
	TXNIP: sh-circ-CUX1 #1+EWSR1 vs. sh-Scb+Mock	5	0.7934	
	TXNIP: sh-circ-CUX1 #2+Mock vs. sh-Scb+Mock	5	0.0288	
	TXNIP: sh-circ-CUX1 #2+EWSR1 vs. sh-Scb+Mock	5	0.8483	
Figure S8C	Wild type: sh-circ-CUX1 #1+Mock vs. sh-Scb+Mock	5	0.0144	One-way ANOVA
	Wild type: sh-Scb+EWSR1 vs. sh-Scb+Mock	5	0.0197	
	Wild type: sh-circ-CUX1 #1+EWSR1 vs. sh-Scb+Mock	5	0.6229	
	Wild type: sh-Scb+MAZ vs. sh-Scb+Mock	5	0.0208	
	Wild type: sh-circ-CUX1 #1+MAZ vs. sh-Scb+Mock	5	0.0613	
	Mutation: sh-circ-CUX1 #1+Mock vs. sh-Scb+Mock	5	0.5962	
	Mutation: sh-Scb+EWSR1 vs. sh-Scb+Mock	5	0.5408	
	Mutation: sh-circ-CUX1 #1+EWSR1 vs. sh-Scb+Mock	5	0.7423	
	Mutation: sh-Scb+MAZ vs. sh-Scb+Mock	5	0.6008	
	Mutation: sh-circ-CUX1 #1+MAZ vs. sh-Scb+Mock	5	0.9533	
Figure S8D	EWSR1: sh-circ-CUX1 #1+Mock vs. sh-Scb+Mock	5	0.8593	One-way ANOVA
	EWSR1: sh-Scb+EWSR1 vs. sh-Scb+Mock	5	0.0141	
	EWSR1: sh-circ-CUX1 #1+EWSR1 vs. sh-Scb+Mock	5	0.0103	
	EWSR1: sh-Scb+MAZ vs. sh-Scb+Mock	5	0.839	
	EWSR1: sh-circ-CUX1 #1+MAZ vs. sh-Scb+Mock	5	0.66	
	MAZ: sh-circ-CUX1 #1+Mock vs. sh-Scb+Mock	5	0.8149	
	MAZ: sh-Scb+EWSR1 vs. sh-Scb+Mock	5	0.6433	
	MAZ: sh-circ-CUX1 #1+EWSR1 vs. sh-Scb+Mock	5	0.8583	
	MAZ: sh-Scb+MAZ vs. sh-Scb+Mock	5	0.0103	
	MAZ: sh-circ-CUX1 #1+MAZ vs. sh-Scb+Mock	5	0.0119	
	CUX1: sh-circ-CUX1 #1+Mock vs. sh-Scb+Mock	5	0.0142	
	CUX1: sh-Scb+EWSR1 vs. sh-Scb+Mock	5	0.0242	
	CUX1: sh-circ-CUX1 #1+EWSR1 vs. sh-Scb+Mock	5	0.7533	
	CUX1: sh-Scb+MAZ vs. sh-Scb+Mock	5	0.0201	
	CUX1: sh-circ-CUX1 #1+MAZ vs. sh-Scb+Mock	5	0.6178	
	S100A9: sh-circ-CUX1 #1+Mock vs. sh-Scb+Mock	5	0.0227	
	S100A9: sh-Scb+EWSR1 vs. sh-Scb+Mock	5	0.0166	
	S100A9: sh-circ-CUX1 #1+EWSR1 vs. sh-Scb+Mock	5	0.5627	
	S100A9: sh-Scb+MAZ vs. sh-Scb+Mock	5	0.0201	
	S100A9: sh-circ-CUX1 #1+MAZ vs. sh-Scb+Mock	5	0.9173	
	MUC4: sh-circ-CUX1 #1+Mock vs. sh-Scb+Mock	5	0.0133	
	MUC4: sh-Scb+EWSR1 vs. sh-Scb+Mock	5	0.0204	
	MUC4: sh-circ-CUX1 #1+EWSR1 vs. sh-Scb+Mock	5	0.4339	
	MUC4: sh-Scb+MAZ vs. sh-Scb+Mock	5	0.0206	
	MUC4: sh-circ-CUX1 #1+MAZ vs. sh-Scb+Mock	5	0.5578	
	KLF10: sh-circ-CUX1 #1+Mock vs. sh-Scb+Mock	5	0.0123	
	KLF10: sh-Scb+EWSR1 vs. sh-Scb+Mock	5	0.0312	
	KLF10: sh-circ-CUX1 #1+EWSR1 vs. sh-Scb+Mock	5	0.8423	
	KLF10: sh-Scb+MAZ vs. sh-Scb+Mock	5	0.0399	
	KLF10: sh-circ-CUX1 #1+MAZ vs. sh-Scb+Mock	5	0.3971	
	TXNIP: sh-circ-CUX1 #1+Mock vs. sh-Scb+Mock	5	0.0205	
	TXNIP: sh-Scb+EWSR1 vs. sh-Scb+Mock	5	0.0289	
	TXNIP: sh-circ-CUX1 #1+EWSR1 vs. sh-Scb+Mock	5	0.9884	
	TXNIP: sh-Scb+MAZ vs. sh-Scb+Mock	5	0.0385	
	TXNIP: sh-circ-CUX1 #1+MAZ vs. sh-Scb+Mock	5	0.8543	
Figure S9A	sh-EWSR1 #1 vs. sh-Scb; IMR32	6	0.0107	One-way ANOVA
	sh-EWSR1 #2 vs. sh-Scb; IMR32	6	0.0123	
	sh-EWSR1 #1 vs. sh-Scb; SH-SY5Y	6	0.0112	
	sh-EWSR1 #2 vs. sh-Scb; SH-SY5Y	6	0.0167	
	sh-MAZ #1 vs. sh-Scb; IMR32	6	0.0105	
	sh-MAZ #2 vs. sh-Scb; IMR32	6	0.0212	
	sh-MAZ #1 vs. sh-Scb; SH-SY5Y	6	0.0123	
	sh-MAZ #2 vs. sh-Scb; SH-SY5Y	6	0.0229	
Figure S9B	Biotin-EIP-22 vs. Biotin-CTLP	5	0.0113	Student's unpaired *t*-test
Figure S9C	Time: EIP-22 vs. CTLP; MCF 10A	5	0.4906	One-way ANOVA
	Concentration: EIP-22 vs. CTLP; MCF 10A	5	0.9864	
	Time: EIP-22 vs. CTLP; SH-SY5Y	5	0.0008	
	Concentration: EIP-22 vs. CTLP; SH-SY5Y	5	0.0032	
	Time: EIP-22 vs. CTLP; SH-SY5Y; 24 hr	5	0.0192	Student's unpaired *t*-test
	Time: EIP-22 vs. CTLP; SH-SY5Y; 48 hr	5	0.0109	
	Time: EIP-22 vs. CTLP; SH-SY5Y; 72 hr	5	0.0203	
	Concentration: EIP-22 vs. CTLP; SH-SY5Y; 5 μM	5	0.0865	
	Concentration: EIP-22 vs. CTLP; SH-SY5Y; 10 μM	5	0.0785	
	Concentration: EIP-22 vs. CTLP; SH-SY5Y; 15 μM	5	0.0373	
	Concentration: EIP-22 vs. CTLP; SH-SY5Y; 20 μM	5	0.0026	
	Concentration: EIP-22 vs. CTLP; SH-SY5Y; 25 μM	5	0.0114	
Figure S9E	CTLP+2-DG vs. CTLP+PBS; IMR32	5	0.0146	One-way ANOVA
	CTLP+3-BP vs. CTLP+PBS; IMR32	5	0.0201	
	EIP-22+PBS vs. CTLP+PBS; IMR32	5	0.0132	
	EIP-22+2-DG vs. CTLP+PBS; IMR32	5	0.0106	
	EIP-22+3-BP vs. CTLP+PBS; IMR32	5	0.0214	
	CTLP+2-DG vs. CTLP+PBS; SH-SY5Y	5	0.0174	
	CTLP+3-BP vs. CTLP+PBS; SH-SY5Y	5	0.0144	
	EIP-22+PBS vs. CTLP+PBS; SH-SY5Y	5	0.0268	
	EIP-22+2-DG vs. CTLP+PBS; SH-SY5Y	5	0.0218	
	EIP-22+3-BP vs. CTLP+PBS; SH-SY5Y	5	0.0102	
Figure S9F	CTLP+2-DG vs. CTLP+PBS; IMR32	5	0.0119	One-way ANOVA
	CTLP+3-BP vs. CTLP+PBS; IMR32	5	0.0237	
	EIP-22+PBS vs. CTLP+PBS; IMR32	5	0.0113	
	EIP-22+2-DG vs. CTLP+PBS; IMR32	5	0.0201	
	EIP-22+3-BP vs. CTLP+PBS; IMR32	5	0.0151	
	CTLP+2-DG vs. CTLP+PBS; SH-SY5Y	5	0.0201	
	CTLP+3-BP vs. CTLP+PBS; SH-SY5Y	5	0.0113	
	EIP-22+PBS vs. CTLP+PBS; SH-SY5Y	5	0.0206	
	EIP-22+2-DG vs. CTLP+PBS; SH-SY5Y	5	0.0121	
	EIP-22+3-BP vs. CTLP+PBS; SH-SY5Y	5	0.0221	
Figure S9G	CTLP+2-DG vs. CTLP+PBS; IMR32	5	0.0112	Student's unpaired *t*-test
	CTLP+3-BP vs. CTLP+PBS; IMR32	5	0.0167	

	EIP-22+PBS vs. CTLP+PBS; IMR32	5	0.0109	One-way ANOVA
	EIP-22+2-DG vs. CTLP+PBS; IMR32	5	0.0204	
	EIP-22+3-BP vs. CTLP+PBS; IMR32	5	0.0123	
	CTLP+2-DG vs. CTLP+PBS; SH-SY5Y	5	0.0204	
	CTLP+3-BP vs. CTLP+PBS; SH-SY5Y	5	0.0144	
	EIP-22+PBS vs. CTLP+PBS; SH-SY5Y	5	0.0222	
	EIP-22+2-DG vs. CTLP+PBS; SH-SY5Y	5	0.0102	
	EIP-22+3-BP vs. CTLP+PBS; SH-SY5Y	5	0.0115	
Figure S10A	Ki-67: EIP-22 vs. CTLP	5	0.0061	Student's unpaired *t*-test
	CD31: EIP-22 vs. CTLP	5	0.0023	
Figure S10C	Glucose: EIP-22 vs. CTLP	5	0.0064	Student's unpaired *t*-test
	Lactate: EIP-22 vs. CTLP	5	0.0092	
	ATP: EIP-22 vs. CTLP	5	0.0059	
Figure S11A	Tumor volume: LV-sh-circ-CUX1 #1 vs. LV-sh-Scb	5	0.0421	One-way ANOVA
	Tumor volume: LV-sh-circ-CUX1 #1 vs. LV-sh-Scb; 1 week	5	0.0637	Student's unpaired *t*-test
	Tumor volume: LV-sh-circ-CUX1 #1 vs. LV-sh-Scb; 2 week	5	0.0129	
	Tumor volume: LV-sh-circ-CUX1 #1 vs. LV-sh-Scb; 3 week	5	0.0201	
	Tumor volume: LV-sh-circ-CUX1 #1 vs. LV-sh-Scb; 4 week	5	0.0131	
	Tumor weight: LV-sh-circ-CUX1 #1 vs. LV-sh-Scb	5	0.0085	
	CUX1 levels: LV-sh-circ-CUX1 #1 vs. LV-sh-Scb	5	0.0076	
Figure S11B	Ki-67: LV-sh-circ-CUX1 #1 vs. LV-sh-Scb	5	0.0028	Student's unpaired *t*-test
	CD31: LV-sh-circ-CUX1 #1 vs. LV-sh-Scb	5	0.0016	
Figure S11D	Glucose: LV-sh-circ-CUX1 #1 vs. LV-sh-Scb	5	0.0065	Student's unpaired *t*-test
	Lactate: LV-sh-circ-CUX1 #1 vs. LV-sh-Scb	5	0.0032	
	ATP: LV-sh-circ-CUX1 #1 vs. LV-sh-Scb	5	0.0084	
Figure S11E	Metastasis: LV-sh-circ-CUX1 #1 vs. LV-sh-Scb	5	< 0.0001	Student's unpaired *t*-test
	Survival: LV-sh-circ-CUX1 #1 vs. LV-sh-Scb	5	0.0064	Log-rank test
