# Supplementary material for: Therapeutic targeting of circ‐CUX1/EWSR1/MAZ axis inhibits glycolysis and neuroblastoma progression
Source: EMBO Mol Med. 2019 Nov 11;11(12):e10835. doi: 10.15252/emmm.201910835 (PMC6895612; doi:10.15252/emmm.201910835)
Supplement: Supplementary file 2 — Expanded View Figures PDF [file EMMM-11-e10835-s002.pdf]

# Expanded View Figures

**Figure EV1. Identification and expression levels of *circ-CUX1*.**

A Flowchart delineating the discovery of *circ-CUX1* from tumor tissues and cell lines. FAM, normal fetal adrenal medulla.
B RT–PCR assay with divergent primers showing the detectable circRNAs of correct size (asterisks) in IMR32 cells.
C Real-time qRT–PCR assay indicating the circRNA levels in FAM ($n$ = 13) and NB tissues ($n$ = 20). Student's $t$-test, *$P < 0.05$ versus FAM.
D Dual-luciferase assay showing transactivation activity of CUX1 in IMR32 and SH-SY5Y cells transfected as indicated ($n$ = 5). Student's $t$-test, one-way ANOVA, *$P < 0.05$ versus empty vector (mock) or scramble shRNA (sh-Scb).
E Real-time qRT–PCR assay indicating *circ-CUX1* levels (normalized to β-actin) in NB tissues ($n$ = 54). Bars are means and whiskers show min to max. Student's $t$-test.
F Kaplan–Meier curve showing overall survival of 54 NB patients with low or high *circ-CUX1* levels (cutoff value = 10.77). Log-rank test.

Data information: Data are presented as mean ± SEM. Exact $P$ values are specified in Appendix Table S4.


© 2019 The Authors

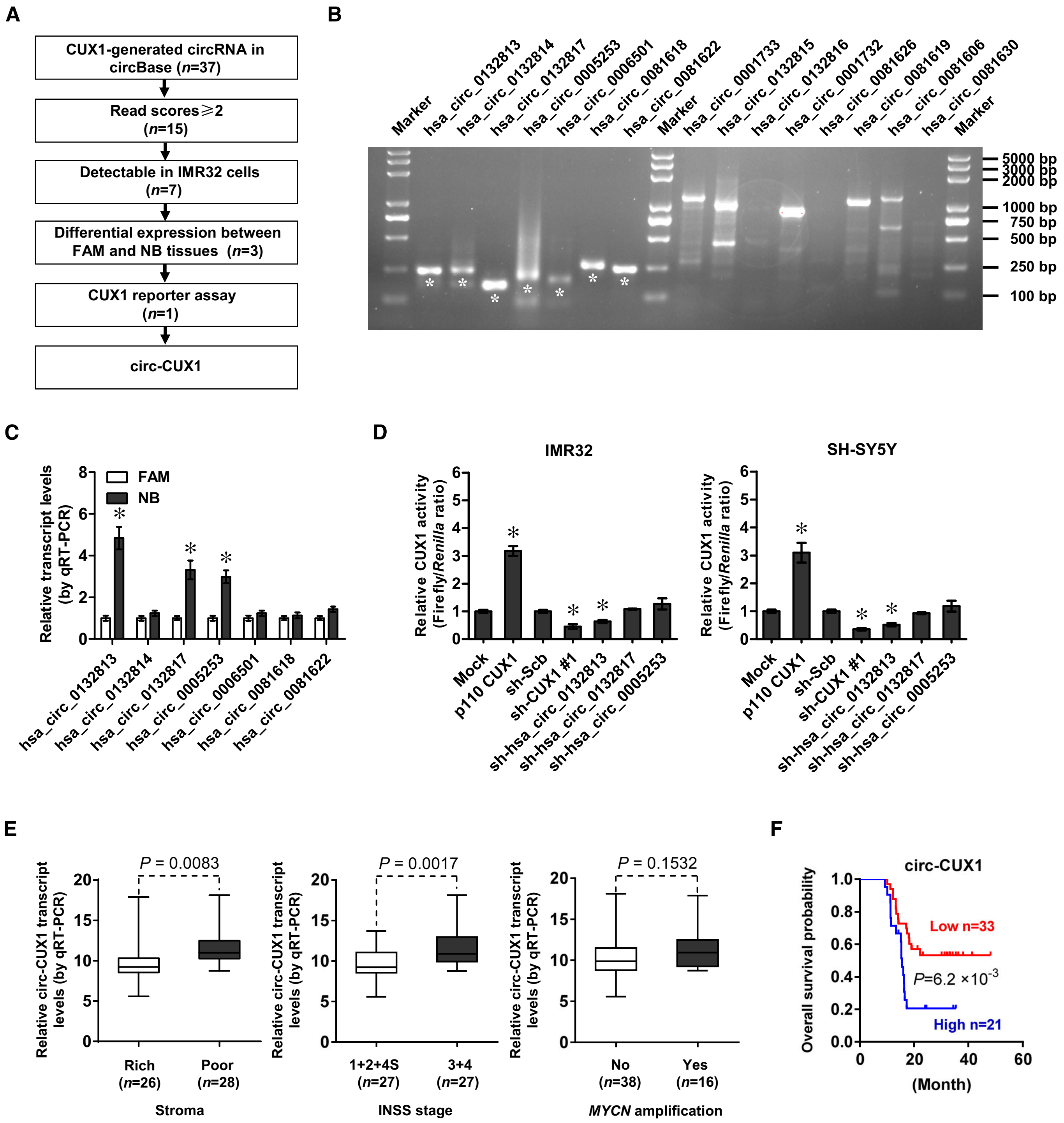
A
CUX1-generated circRNA in circBase (n=37)
Read scores⩾2 (n=15)
Detectable in IMR32 cells (n=7)
Differential expression between FAM and NB tissues (n=3)
CUX1 reporter assay (n=1)
circ-CUX1
B
Marker
hsa_circ_0132813
hsa_circ_0132814
hsa_circ_0132817
hsa_circ_0005253
hsa_circ_0006501
hsa_circ_0081618
hsa_circ_0081622
Marker
hsa_circ_0001733
hsa_circ_0132815
hsa_circ_0132816
hsa_circ_0001732
hsa_circ_0081626
hsa_circ_0081619
hsa_circ_0081606
hsa_circ_0081630
Marker
5000 bp
3000 bp
2000 bp
1000 bp
750 bp
500 bp
250 bp
100 bp
C
Relative transcript levels (by qRT-PCR)
FAM
NB
D
IMR32
SH-SY5Y
Relative CUX1 activity (Firefly/Renilla ratio)
Mock
p110 CUX1
sh-Scb
sh-CUX1 #1
sh-hsa_circ_0132813
sh-hsa_circ_0132817
sh-hsa_circ_0005253
E
Relative circ-CUX1 transcript levels (by qRT-PCR)
P = 0.0083
Rich (n=26)
Poor (n=28)
Stroma
P = 0.0017
1+2+4S (n=27)
3+4 (n=27)
INSS stage
P = 0.1532
No (n=38)
Yes (n=16)
MYCN amplification
F
circ-CUX1
Overall survival probability
Low n=33
P=6.2 ×10-3
High n=21
(Month)

Figure EV1.

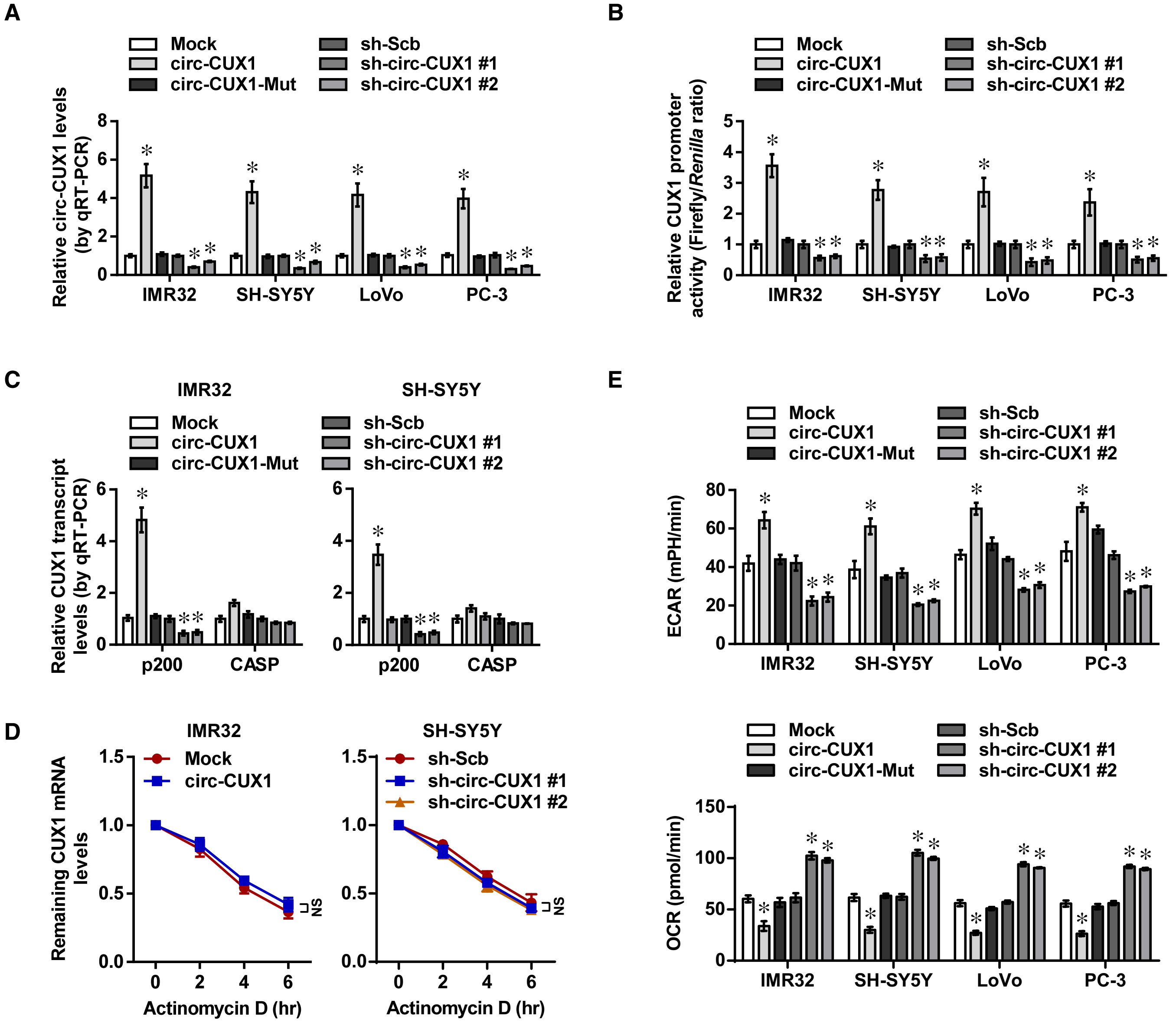


**Figure EV2.** ***Circ-CUX1*** **facilitates** ***CUX1*** **transcription and aerobic glycolysis of tumor cells.**

A Real-time qRT–PCR assay indicating expression of *circ-CUX1* (normalized to β-actin) in IMR32, SH-SY5Y, LoVo, or PC-3 cells stably transfected as indicated ($n = 5$). One-way ANOVA, *$P < 0.05$ versus empty vector (mock) or scramble shRNA (sh-Scb).

B Dual-luciferase assay showing the activity of *CUX1* promoter in IMR32, SH-SY5Y, LoVo, or PC-3 cells stably transfected as indicated ($n = 5$). One-way ANOVA, *$P < 0.05$ versus mock or sh-Scb.

C Real-time qRT–PCR assay indicating expression of *CUX1* isoforms (normalized to β-actin) in IMR32 and SH-SY5Y cells stably transfected as indicated ($n = 5$). One-way ANOVA, *$P < 0.05$ versus empty vector (mock) or scramble shRNA (sh-Scb).

D Real-time qRT–PCR assay indicating *CUX1* mRNA levels in IMR32 and SH-SY5Y cells stably transfected as indicated ($n = 5$), and those treated with actinomycin D (5 μg ml$^{-1}$). One-way ANOVA, NS: non-significant.

E Seahorse extracellular flux assay showing ECAR and OCR in IMR32, SH-SY5Y, LoVo, or PC-3 cells stably transfected as indicated ($n = 6$). One-way ANOVA, *$P < 0.05$ versus mock or sh-Scb.

Data information: Data are presented as mean ± SEM. Exact *P* values are specified in Appendix Table S4.


© 2019 The Authors
