# Supplementary material for: Therapeutic targeting of circ‐CUX1/EWSR1/MAZ axis inhibits glycolysis and neuroblastoma progression
Source: EMBO Mol Med. 2019 Nov 11;11(12):e10835. doi: 10.15252/emmm.201910835 (PMC6895612; doi:10.15252/emmm.201910835)
Supplement: Supplementary file 3 — Movie EV1 [file EMMM-11-e10835-s003.zip › EMM201910835V2_Movie_EV1/EMM201910835V2_Movie_EV1.doc]

**Movie EV1 - 3D reconstruction of co-localization of *circ-CUX1* (red) and EWSR1 (green)** analyzed by dual RNA-FISH and immunofluorescence staining assay in IMR32 cells stably transfected with empty vector (mock) or *circ-CUX1*.
